# Left Ventricle Unloading with Veno-Arterial Extracorporeal Membrane Oxygenation for Cardiogenic Shock. Systematic Review and Meta-Analysis

**DOI:** 10.3390/jcm9041039

**Published:** 2020-04-07

**Authors:** Mariusz Kowalewski, Pietro Giorgio Malvindi, Kamil Zieliński, Gennaro Martucci, Artur Słomka, Piotr Suwalski, Roberto Lorusso, Paolo Meani, Antonio Arcadipane, Michele Pilato, Giuseppe Maria Raffa

**Affiliations:** 1 Clinical Department of Cardiac Surgery, Central Clinical Hospital of the Ministry of Interior and Administration, Centre of Postgraduate Medical Education, 02607 Warsaw, Poland; kowalewskimariusz@gazeta.pl (M.K.); suwalski.piotr@gmail.com (P.S.); 2 Department of Cardio-Thoracic Surgery, Heart and Vascular Centre, Maastricht University Medical Centre, 6229 Maastricht, The Netherlands; roberto.lorussobs@gmail.com; 3 Thoracic Research Centre, Nicolaus Copernicus University, Collegium Medicum in Bydgoszcz, Innovative Medical Forum, 85067 Bydgoszcz, Poland; 4 Wessex Cardiothoracic Centre, University Hospital Southampton, Southampton SO16 6YD, UK; pg.malvindi@hotmail.com; 5 Student Scientific Society, Warsaw Medical University, 02091 Warsaw, Poland; kamilziel@gmail.com; 6 Anesthesia and Intensive Care Department, IRCCS-ISMETT, 90127 Palermo, Italy; gmartucci@ismett.edu (G.M.); aarcadipane@ismett.edu (A.A.); 7 Chair and Department of Pathophysiology, Nicolaus Copernicus University, Collegium Medicum in Bydgoszcz, 85067 Bydgoszcz, Poland; artur.slomka@cm.umk.pl; 8 Cardiovascular Research Institute Maastricht (CARIM), Maastricht University, 6229 Maastricht, The Netherlands; 9 Department of Intensive Care Unit, Maastricht University Medical Centre (MUMC+), 6229 Maastricht, The Netherlands; paolo.meani@ospedaleniguarda.it; 10 Department for the Treatment and Study of Cardiothoracic Diseases and Cardiothoracic Transplantation, IRCCS-ISMETT (Instituto Mediterraneo per i Trapianti e Terapie ad alta specializzazione), 90127 Palermo, Italy; mpilato@ISMETT.edu

**Keywords:** cardiogenic shock, extracorporeal membrane oxygenation, extracorporeal life support, resuscitation

## Abstract

During veno-arterial extracorporeal membrane oxygenation (VA-ECMO), the increase of left ventricular (LV) afterload can potentially increase the LV stress, exacerbate myocardial ischemia and delay recovery from cardiogenic shock (CS). Several strategies of LV unloading have been proposed. Systematic review and meta-analysis in accordance with the Preferred Reporting Items for Systematic Reviews and Meta-Analyses (PRISMA) statement included adult patients from studies published between January 2000 and March 2019. The search was conducted through numerous databases. Overall, from 62 papers, 7581 patients were included, among whom 3337 (44.0%) received LV unloading concomitant to VA-ECMO. Overall, in-hospital mortality was 58.9% (4466/7581). A concomitant strategy of LV unloading as compared to ECMO alone was associated with 12% lower mortality risk (RR 0.88; 95% CI 0.82–0.93; *p* < 0.0001; *I*^2^ = 40%) and 35% higher probability of weaning from ECMO (RR 1.35; 95% CI 1.21–1.51; *p* < 0.00001; *I*^2^ = 38%). In an analysis stratified by setting, the highest mortality risk benefit was observed in case of acute myocardial infarction: RR 0.75; 95%CI 0.68–0.83; *p* < 0.0001; *I*^2^ = 0%. There were no apparent differences between two techniques in terms of complications. In heterogeneous populations of critically ill adults in CS and supported with VA-ECMO, the adjunct of LV unloading is associated with lower early mortality and higher rate of weaning.

## 1. Introduction

Veno-arterial extracorporeal membrane oxygenation (VA-ECMO) provides life support for patients with refractory cardiogenic shock and significantly improves their survival working as a bridge to either recovery or other long-term treatments [1,2,3,4,5,6,7,8,9].

A well recognized limitation of the retrograde aortic flow while on VA-ECMO is the increase of left ventricular (LV) afterload [10], which can potentially lead to high LV stress and may exacerbate myocardial ischemia thus delaying recovery from cardiogenic shock [11]. Elevated LV pressure can also promote LV dilatation and trigger ventricular arrhythmias, or, secondarily, increase left atrial pressure causing pulmonary edema [12]. Ultimately, a reduced flow across the aortic valve can induce formation of thrombus in the LV or the aortic root [13].

Several LV unloading strategies have been described and proposed in order to minimize the risk of these complications [14], however, the available evidences are still conflicting whether these techniques are safe and useful adjuncts to VA-ECMO in patients with cardiogenic shock [15,16,17,18].

The aim of this study was to comprehensively assess the impact on early outcomes of different strategies of LV unloading in patients undergoing VA-ECMO and sustaining advanced cardiogenic shock by various etiologies.

## 2. Experimental Section

### 2.1. Data Sources and Search Strategy

This systematic review and meta-analysis was performed in accordance with the Preferred Reporting Items for Systematic Reviews and Meta-Analyses (PRISMA) [19]. The PRISMA checklist is available as Appendix A Table A1. Research of relevant studies was limited to the period January 2000–March 2019, through PubMed, EMBASE, CINAHL, the Cochrane Register of Controlled Clinical Trials (CENTRAL) and Google Scholar. Abstracts were eligible for detailed assessment if available online and reporting outcomes of interest. The search terms were: “extracorporeal membrane oxygenation” and “extracorporeal life support”. No language restrictions were imposed. References of original articles were reviewed manually and cross-checked for other relevant reports. Authors of individual studies were contacted for missing data.

### 2.2. Selection Criteria and Quality Assessment

Human studies were included if they assessed survival after VA-ECMO or weaning from VA-ECMO support instituted for refractory cardiogenic shock. Research centers were checked to avoid potential overlapping patients and those reporting on smaller samples of patients were excluded. Reviews and case reports were not considered. Two independent reviewers (M.K. and K.Z.) selected the studies for inclusion, extracted studies, as well as patient characteristics of interest and relevant outcomes. Two authors (M.K. and K.Z.) independently assessed the trials’ eligibility and risk of bias. Risk of bias at the individual study level was assessed using the Risk of Bias in Not-randomized Studies-of Interventions (ROBINS-I) tool [20]. Any divergences were resolved by a third reviewer (G.R.) and quantified using the approach of Cohen’s kappa [21].

### 2.3. Endpoint Selection

The primary endpoints were in-hospital/30-day survival and weaning from VA-ECMO. Secondary endpoints were in-hospital cerebrovascular events (CVE), brain death, limb complications, reoperation for bleeding, sepsis and acute kidney failure w/wo continuous veno-venous hemofiltration (CVVH). Outcome definitions were the ones adopted by the investigators of the included studies.

### 2.4. Statistical Analysis

Statistical analyses were performed in Comprehensive Meta-Analysis, v. 2.0 (Biostat, Englewood, NJ, USA) and Review Manager 5.3 (The Nordic Cochrane Centre, Copenhagen, Denmark). The results are expressed as pooled untransformed proportion risk ratios (RR) with their 95% confidence intervals (CI). Heterogeneity across studies was evaluated using the *I*^2^ test. To control for the anticipated heterogeneity among observational studies, absolute values and means were pooled using inverse variance random effects models. The primary endpoints were assessed in relation to the specific setting according to etiology of cardiogenic shock which included: (1) postcardiotomy shock (PCS), (2) acute myocardial infarction (AMI), (3) myocarditis and (4) mixed cohort of different etiologies including both postcardiotomy shock and AMI and other etiologies. Number needed to treat (NNT) was calculated for these subgroups. Secondary analysis focused on specific left ventricular unloading strategy: (1) intra-aortic balloon pump (IABP), (2) LV venting with cannula in left atrium or ventricle or (3) percutaneous ventricular assist device (Impella, Abiomed, Danvers, MA, USA). We performed separate analysis of studies with propensity score matching or presenting propensity score adjusted odds ratio (OR) of primary endpoints. We investigated if use of different unloading strategies had influence on complication rates, ECMO duration and weaning rates by means of meta-regression analyses [22]. Similarly, we addressed the impact of hypertension, diabetes, age and gender on mortality outcome. Sensitivity analyses were performed by excluding from analyses single studies, one at a time, and repeating the calculations. Publication bias was assessed (1) by visual approach plotting log event rate against standard error in the funnel plot; and (2) by linear regression approach [23].

## 3. Results

### 3.1. Study Selection

**Figure 1:**
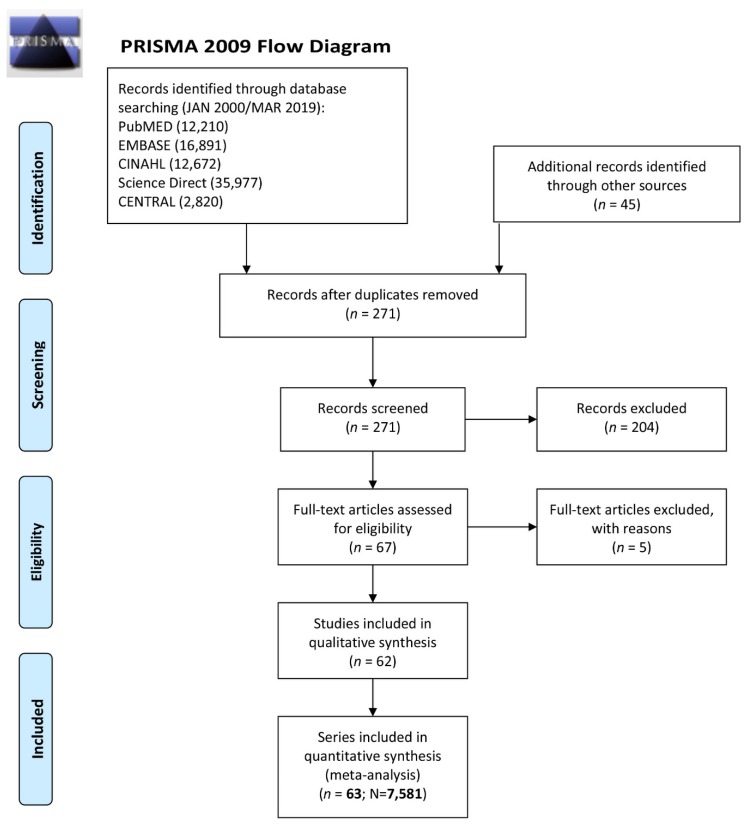
Preferred Reporting Items for Systematic Reviews and Meta-Analyses (PRISMA) flow diagram of study selection process. References of included and excluded studies are listed in the supplementary material.

The study selection process and reasons for exclusion of some studies are described in Figure 1. A systematic search of the online databases allowed us to screen based on title and collect 271 potentially eligible records that were retrieved for scrutiny. Of those, 204 were further excluded because they were not pertinent to the design of the meta-analysis or did not meet the explicit inclusion criteria based on their content. To avoid potential double inclusion of patients’ populations, 5 studies were excluded (Supplementary Material: Part 1) since they were conducted in the same institution in overlapping time frames. Sixty-three series of patients from 62 observational studies (Supplementary Material: Part 2) that enrolled 7581 patients eventually were included in the analysis. Patients were divided into 2 groups: those undergoing LV unloading concomitant to VA-ECMO and those undergoing VA-ECMO alone; (3337; 44.0%) vs. (4244; 56.0%).

Patients undergoing VA-ECMO had a mean age of 57.8 years and 71.0% were male. Follow-up across the studies varied between 30-day and in-hospital survival. Table A2 details about studies and Table A3 about patients’ characteristics. Risk of bias for each study across each of the seven risk of bias domains is presented in Table A4. Overall, the studies reported either moderate or serious risk of bias. Given the overall high risk of bias along with the limited number of studies, all articles were retained for the purposes of this review. Most commonly, biases arose from (1) selection of participants for the study, and (2) subjective distribution of the participants within the study arms.

Populations included patients on VA-ECMO support for cardiogenic shock secondary to mixed etiologies (23 series, 4204 patients), PCS (22 series, 2324 patients) and AMI (14 series, 950 patients); VA-ECMO was employed for myocarditis in 4 series enrolling 103 patients.

### 3.2. Primary Endpoints

#### 3.2.1. Mortality

**Figure 2:**
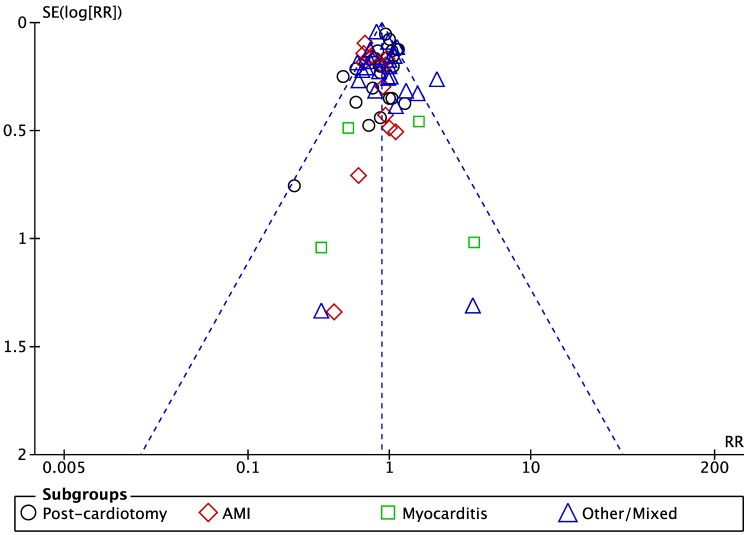
Publication bias analysis (SE: standard error).

**Figure 3:**
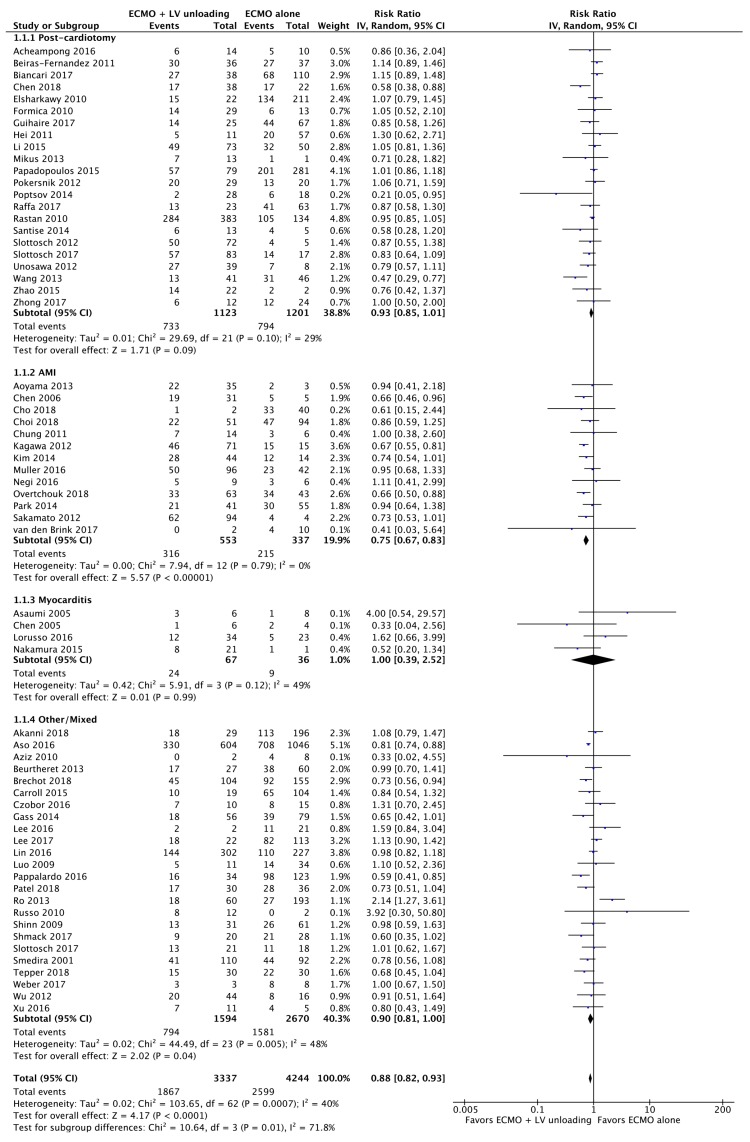
All-cause in-hospital mortality rate for patients receiving extracorporeal membrane oxygenation (ECMO) + left ventricular (LV) unloading versus ECMO alone treatment according to cardiogenic shock etiology.

All 63 included series (7581) contributed to the analysis of overall mortality; constructed funnel plot did not reveal any signs of publication bias or big study effect (Figure 2): overall in-hospital mortality was 58.9% (4466/7581). LV unloading as adjunct to ECMO support was associated with 12% lower risk of mortality compared to ECMO alone therapy: risk ratio (RR); 95% confidence intervals (CIs): 0.88 (0.82–0.93); *p < 0*.0001; *I*^2^ = 40%; Figure 3.

**Figure A1:**
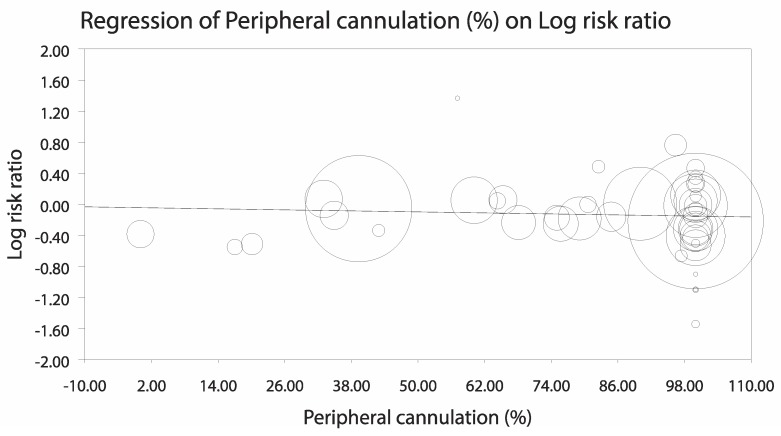
Meta-regression showing the impact of the rate of peripheral cannulation on Log odds ratio.

**Figure A2:**
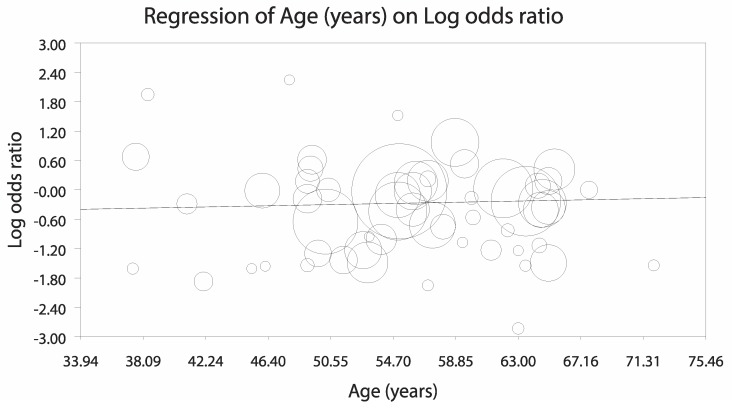
Meta-regression showing the impact of age distribution on Log odds ratio.

**Figure A3:**
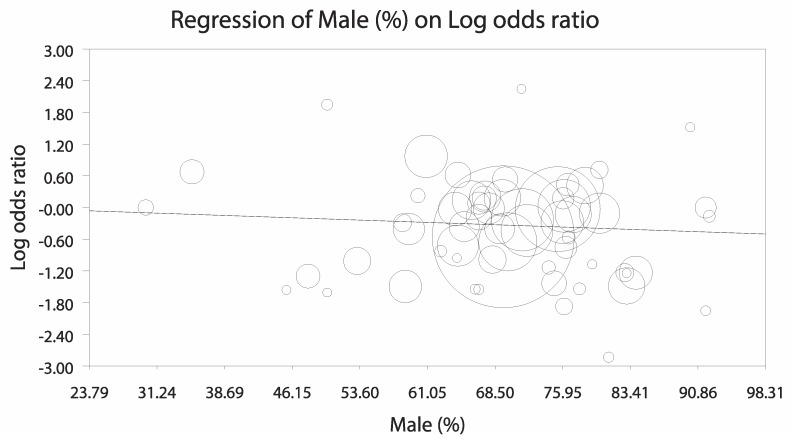
Meta-regression showing the impact of gender distribution on Log odds ratio.

**Figure A4:**
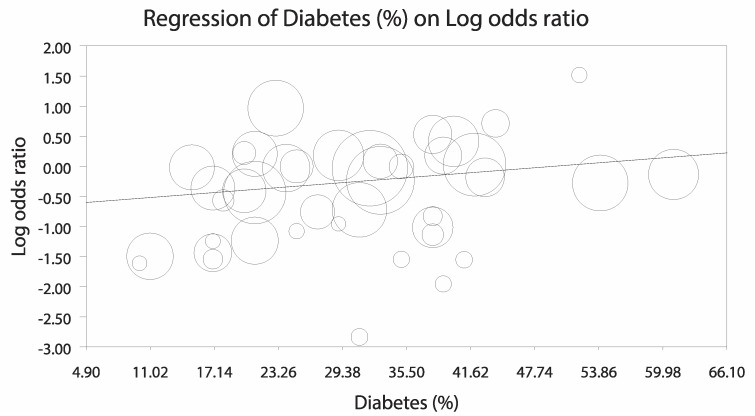
Meta-regression showing the impact of diabetes percentage distribution on Log odds ratio.

**Figure A5:**
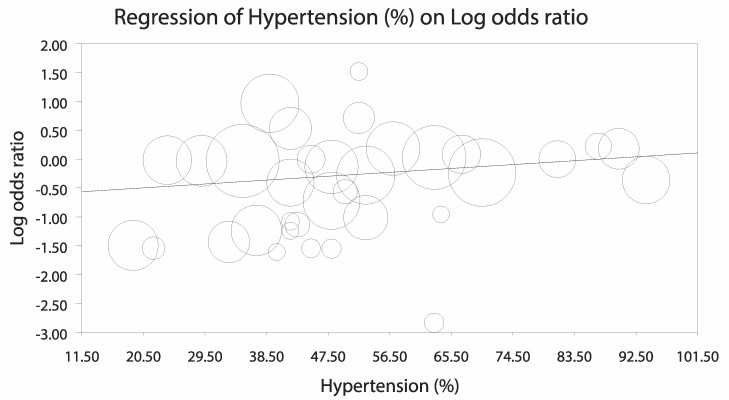
Meta-regression showing the impact of hypertension distribution on Log odds ratio.

The highest mortality risk benefit (25%) was observed in the subgroup of patients undergoing LV unloading + ECMO for AMI: RR (95%CIs): 0.75 (0.67–0.83); *p < 0*.00001; *I*^2^ = 0%; NNT = 15. A mortality risk benefit of 11% was demonstrated in studies including mixed indication for LV unloading + ECMO: RR (95%CIs): 0.90 (0.81–1.00); *p* = 0.04; *I*^2^ = 48%; NNT = 11; In patients with postcardiotomy cardiogenic shock, LV unloading on top of ECMO was associated with 7% non-significantly lower mortality risk; RR (95%CIs): 0.93 (0.85–1.01); *p* = 0.09; *I*^2^ = 29%; NNT = 125. No differences were seen between LV-unloading + ECMO as compared to ECMO alone in patients with myocarditis; NNt = 9. Significant statistical differences as of extent of benefit were demonstrated between subgroups (*p*_interaction_ = 0.01). No impact on early mortality was found according to the type of cannulation, peripheral and central, in a meta-regression analysis, Figure A1. Similarly, these were unaffected by age, gender, diabetes and hypertension status (Figure A2, Figure A3, Figure A4 and Figure A5).

#### 3.2.2. Weaning

**Figure 4:**
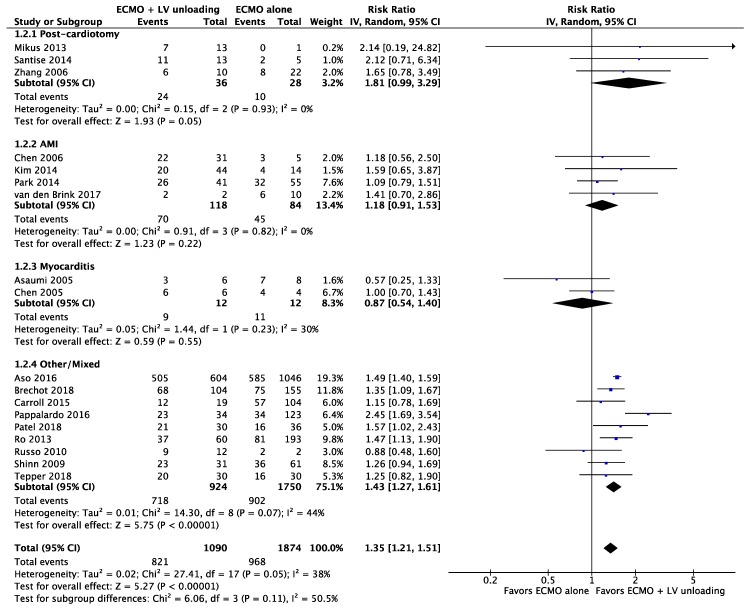
Weaning rate for patients receiving ECMO + LV unloading vs. ECMO alone treatment according to cardiogenic shock etiology.

Seventeen studies with nearly 3000 patients reported on weaning rates in subsets receiving LV unloading + ECMO as compared to ECMO therapy alone. In the overall analysis, LV unloading was associated with 35% higher probability of weaning from ECMO: RR (95%CIs): 1.35 (1.21–1.51); *p <* 0.00001; *I*^2^ = 38%: weaning was possible in 60.4% (1789/2964) of included patients with corresponding rates of 75.3% (821/1090) and 51.7% (968/1874) for LV unloading + ECMO and ECMO alone; Figure 4. LV unloading on top of ECMO was associated with a higher chance of weaning in postcardiotomy cardiogenic shock: RR (95%CIs): 1.81 (0.99–3.29); *p* = 0.05; *I*^2^ = 0%. Differences between subgroups were not statistically significant.

### 3.3. Secondary Endpoints

**Figure A6:**
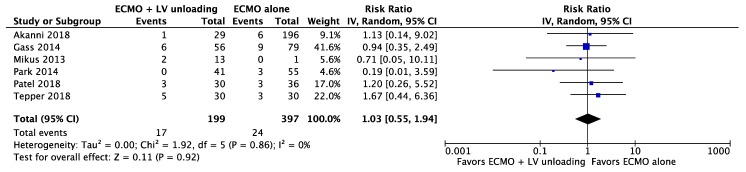
Neurologic complications.

**Figure A7:**
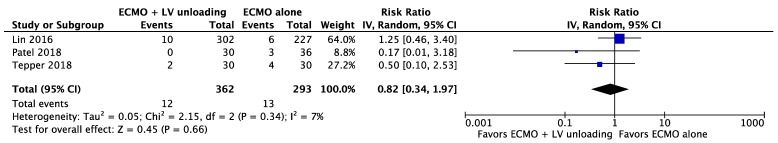
Brain death.

**Figure A8:**
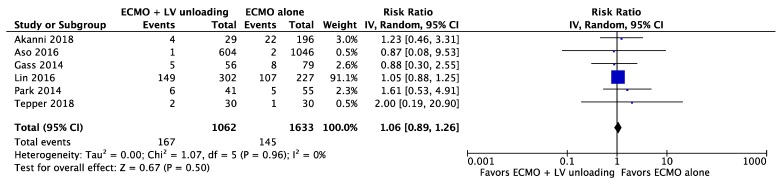
Limb complications.

**Figure A9:**
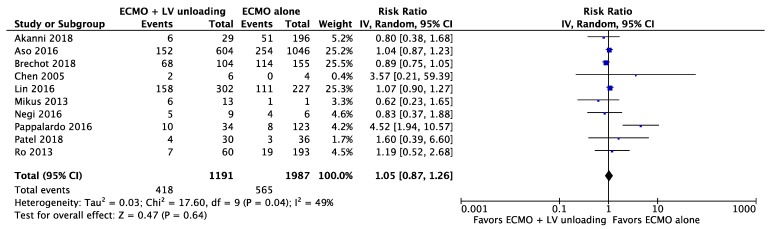
Acute kidney injury (AKI).

**Figure A10:**
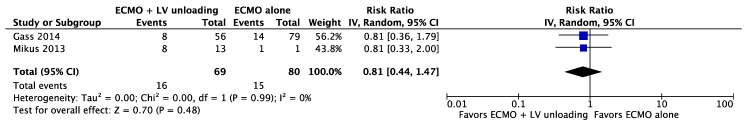
Revision for bleeding.

**Figure A11:**
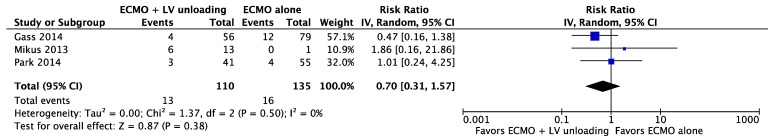
Sepsis.

**Figure A12:**
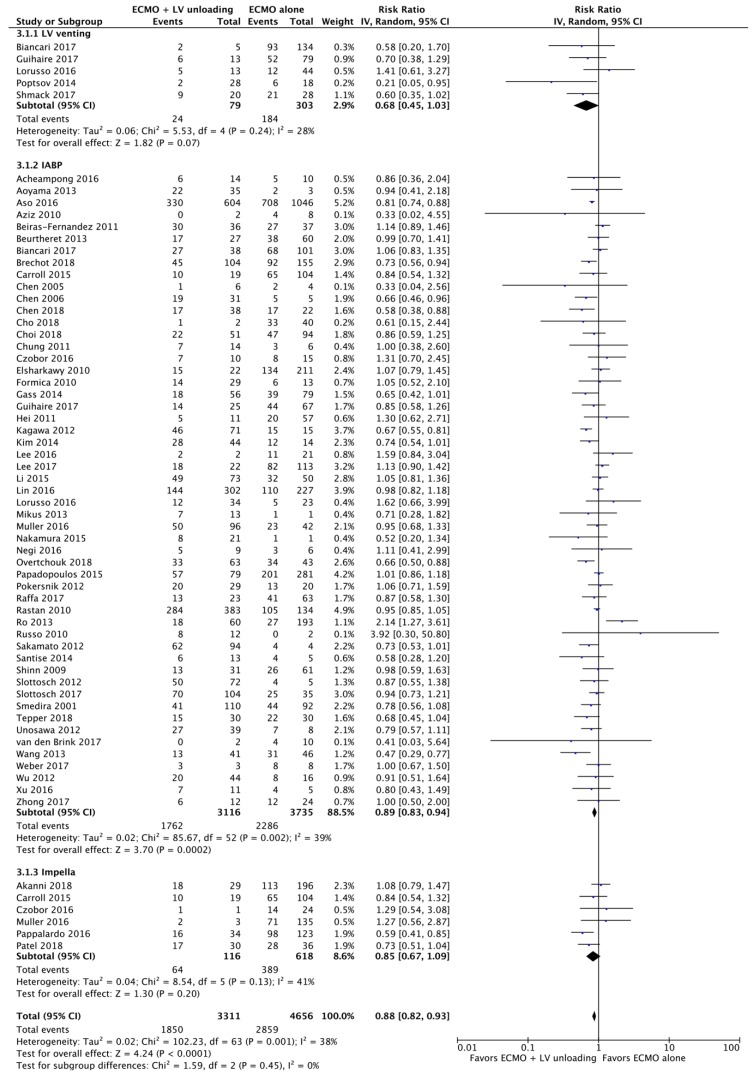
Mortality by device.

**Figure A13:**
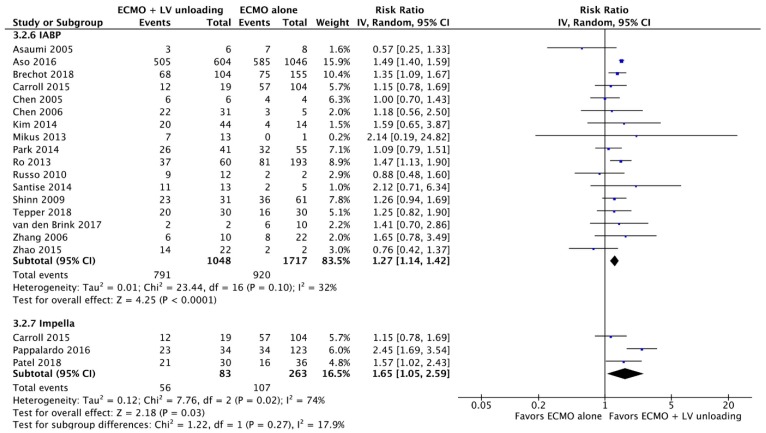
Weaning by device.

There were no apparent differences between LV unloading + ECMO vs. ECMO alone treatment regarding the secondary endpoints (Figure A6, Figure A7, Figure A8, Figure A9, Figure A10, Figure A11, Figure A12 and Figure A13). Neurologic complications incidence was reported in 6 studies (596 patients) with respective 8.5% (17/199) vs. 6.0% (24/397) for ECMO + LV unloading vs. ECMO alone (RR (95%CIs): 1.03 (0.55–1.94); *p* = 0.92; *I*^2^ = 0%); Figure A6. Similarly, non-significant differences in terms of brain death was seen: (RR (95%CIs): 0.82 (0.34–1.97); *p* = 0.66; *I*^2^ = 7%; Figure A7. ECMO + LV unloading was not associated with any benefit nor harm in analysis of: limb complications (6 studies; 2695 patients): RR (95%CIs): 1.06 (0.89–1.26); *p* = 0.50; *I*^2^ = 0% (Figure A8); acute kidney injury (10 studies; 3178 patients): RR (95%CIs): 1.03 (0.87–1.26); *p* = 0.64; *I*^2^ = 49% (Figure A9); revision for bleeding: RR (95%CIs): 0.81 (0.44–1.47); *p* = 0.48; *I*^2^ = 0% (Figure A10); sepsis: RR (95%CIs): 0.70 (0.31–1.57); *p* = 0.38; *I*^2^ = 0% (Figure A11).

#### 3.3.1. Analysis Stratified by LV Unloading Technique

As secondary analysis, we assessed the impact of the different unloading techniques on mortality and weaning: 5 studies (382 patients) reported on LV unloading by direct LV venting catheters: a statistical trend of 32% reduced mortality risk was demonstrated for ECMO + LV venting as compared to ECMO alone: RR (95%CIs): 0.68 (0.45–1.03); *p* = 0.07; *I*^2^ = 28%; Figure A12 and Table A5. Respective mortality rates were 30.4% (24/79) vs. 60.7% (184/303) for LV unloading + ECMO and ECMO alone. No data was available about the rate of weaning in the groups receiving an LV venting. Use of IABP as an adjunct to ECMO was assessed in 56 studies (7015 patients): mortality rates were 56.4% (1791/3174) and 60.7% (2331/3841) for ECMO + IABP vs. ECMO alone; RR (95%CIs): 0.89 (0.84–0.95); *p* = 0.0004; *I*^2^ = 39%. Intra-aortic balloon pump was further associated with significant increased chance of weaning from ECMO: RR (95%CIs): 1.27 (0.14–1.42); *p < 0*.0001; *I*^2^ = 32%); Figure A13. Lower, yet statistically non-significant mortality risk was found for ECMO + Impella as compared to ECMO alone (6 studies; 734 patients): RR (95%CIs): 0.85 (0.67–1.09); *p* = 0.20; *I*^2^ = 41%. Additionally, Impella device was independently associated with higher chance of weaning from ECMO: RR (95%CIs): 1.65 (1.05–2.59); *p* = 0.03; *I*^2^ = 74% (Figure A13).

#### 3.3.2. Sensitivity Analyses

**Figure 5:**
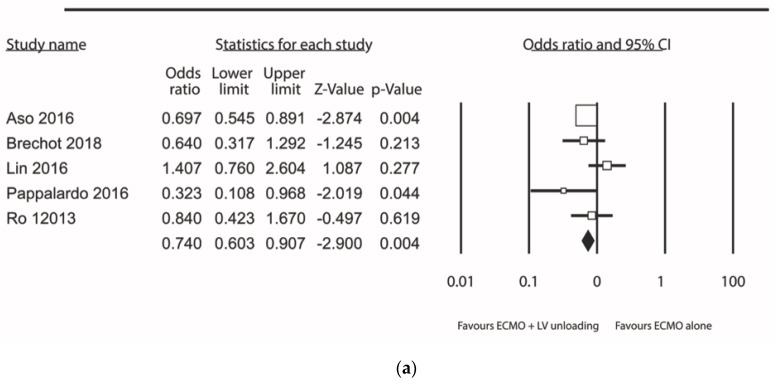

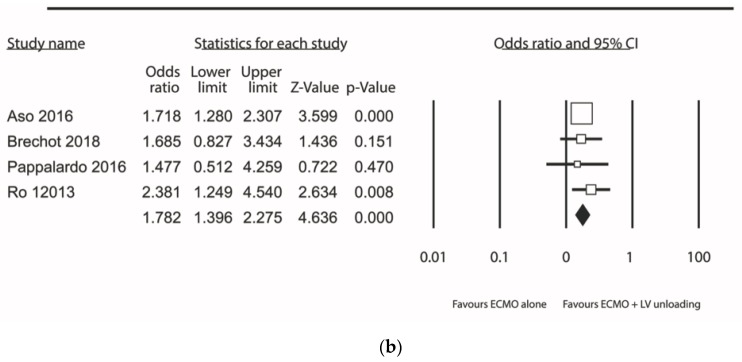
All-cause in-hospital mortality rate (a) and weaning rate (b) from studies reporting propensity adjusted results.

Analyses were repeated as sensitivity for primary endpoints mortality and weaning from ECMO this time included only studies that reported effect estimates for propensity matched cohorts only: 5 studies (Supplementary Material: Part 3) provided propensity adjusted estimates of mortality; pooled together, LV unloading on top of ECMO was associated with over 25% statistically significant reduction in the odds of mortality as compared to ECMO alone: OR (95%CIs): 0.74 (0.60–0.91); *p* = 0.004; *I*^2^ = 42%; Figure 5a.

Weaning rates for comparison LV unloading + ECMO and ECMO alone adjusted for propensity were reported in 4 studies (Supplementary Material: Part 4); again, LV unloading on top of ECMO was associated with over 75% significantly higher odds to wean from ECMO: OR (95%CIs): 1.78 (1.40–2.28); *p < 0*.001; *I*^2^ = 0%; Figure 5b.

Sensitivity analyses performed by deleting each study, one at a time, and repeating the calculations did not change the direction nor magnitude of the treatment effect, suggesting absence of big-study effect.

## 4. Discussion

VA-ECMO is an established treatment able to provide a mechanical circulatory support for patients in cardiogenic shock, aiming a bridge to decision or to myocardial recovery [1,2,3,4,5,6,7,8,9]. Improvements in technology have mitigated the interaction between artificial surfaces of ECMO circuits and blood [24]. However, other adverse effects, known as “flow-related dynamic”, are strictly associated, both in central and peripheral ECMO configuration, with the retrograde direction of the flow towards a dysfunctioning left ventricle. Two major issues have been longer debated by the scientific community: the first is the difference in outcomes and hemodynamic support between the central and peripheral cannulation; the second is the clinical impact of the left ventricle unloading and the strategy to achieve a safe and effective ventricular decompression. The first issue has been already addressed by our group [25]; aim of the current meta-analysis is to address the question whether myocardial unloading is beneficial or, by raising the complexity of ECMO management, futile or potentially detrimental to patients’ outcomes.

ECLS institution increases the left ventricle afterload with a rise in LV end-systolic volume and reduction in LV stroke volume. If peripheral resistance and LV contractility are fixed, increase in LV end-diastolic volume is the only way to overcome the afterload via the Frank–Starling mechanism. In this case, higher levels of VA-ECMO flow cause a progressive rise in LV end-diastolic pressure, LA pressure, pulmonary capillary wedge pressure, that are associated with a further reduced LV stroke volume [26,27,28]. High afterload situations with inability of LV to manage the transpulmonary blood flow, inadequate response to inotropes, complete cardiac arrest with incomplete venous drainage and aortic valve incompetence are the commonest risk factors for LV distension. Patients with severely impaired LV function and/or right ventricular dysfunction are more prone to develop an ineffective LV unloading [29]. LV overload increases wall stress, myocardial oxygen consumption and induce sub-endocardial ischemia and ventricular arrhythmias, jeopardizing ventricular recovery particularly in the presence of ischemia-induced myocardial impairment. The consequence of the pressure overload may ultimately account for pulmonary congestion and edema.

If the overload is extreme and LV contractile impairment significant, the LV is unable to provide a sufficient flow against the increased afterload and the aortic valve may remain closed even during systole, causing blood stasis in the left ventricle, left atrium and aorta, and accounting for intracardiac thrombosis which has been reported in up to 6% of the cases [30,31]. The LV dilatation may further induce annular dilatation and mitral valve leaflet tethering with severe functional regurgitation, thus, particularly in in patients with a history of chronic heart failure and LV dysfunction with a dilated LV, worsening the pulmonary congestion [32].

Definition of LV distension during VA-ECMO is lacking in the literature. Truby et al. [33] attempt to classify and grade the LVD according to the evidence of pulmonary edema on chest radiography and increased pulmonary artery diastolic blood pressure (>25 mmHg). The latter was a surrogate of the wedge pressure evaluated in the “Should we emergently revascularize occluded coronaries for cardiogenic shock” (SHOCK) trial [34]. Clinical evidence of LV distension requiring immediate decompression was inversely related to the chance of myocardial recovery. Meani et al. [32] defined and graded the severity of LV loading during VA-ECMO according to hemodynamic parameters, chest X-ray and echocardiogram findings.

These differences in definitions and assessments may account for the high variability of LV distension rate in the literature. Camboni et al. [35] reported need for LV decompression in 2% of the cases in more than 600 patients. A strict and longer afterload reduction (> 24 hours), targeted lower ECMO flow and a restrictive fluid management were the strategy adopted in this large series. In Truby et al. [33] the clinical and subclinical (not warranting immediate decompression) LV distension occurred in 7% and 22% of patients, respectively. Among 184 peripheral VA ECMO in the series of Meani et al. [36], 5.4% required IABP placement because of a protracted closure of the aortic valve.

Drugs administration is the first line treatment of left ventricle distension. Inotropes can be administered to increase LV contractility while vasodilators may reduce the peripheral resistances and decrease left ventricle afterload. A careful fluid balance (diuretics/fluid restriction) avoiding fluid overload can reduce the risk of pulmonary edema. Ventilatory optimization, including higher PEEP, prolonged expiration time and lower tidal volume, may further improve the venous drainage.

**Figure 6:**
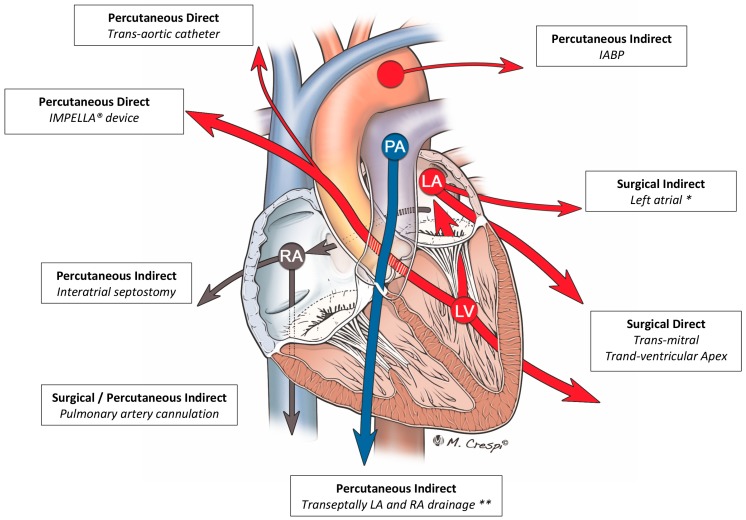
Left ventricle unloading strategies classified according to the direct or indirect, percutaneous or surgical strategies. The differences in arrows’ width is intended suggesting the efficacy of left ventricle unloading (greater for direct surgical approach and Impella device). The color of the dash is intended suggesting blood oxygenation. Further techniques, not included in the picture, are the direct LV transaortic device by PulseCath device, percutaneous indirect LA drainage with TandemHeart transeptal cannula. PA: pulmonary artery; LA: left atrium; LV: left ventricle; RA: right atrium; * achieved through right superior pulmonary vein, left atrial roof, interatrial groove; ** simultaneous left and right atrial drainage with the multistage cannula coming from the femoral vein and positioned transeptally.

When medical treatment is not successful, the non-pharmacological management of LV distension, acting with a “direct” or indirect” mechanism, can be obtained through a surgical or percutaneous strategy (Figure 6).

### 4.1. IABP

IABP has been the most used technique to unload the left ventricle during ECMO support [37]. The IABP acts with several “indirect” mechanisms reducing both the LV afterload (enhanced systolic ejection) and the LV end-diastolic pressure (enhanced left atrial and pulmonary venous unloading). The IABP induces the aortic valve opening [36], improves coronary and abdominal circulation [38], allows pulsatility in end organ capillary bed [39], it is easy to implant and has contained costs. In animal studies the role of counterpulsation in VA-ECMO support seems controversial. Zobel [40] and Sauren [41] showed that IABP has beneficial effects on LV performance. Instead, Belohlávek et al. [42] showed that the combination of femoral VA-ECMO and IABP could impair coronary perfusion. In clinical practice the combination ECMO/IABP was associated with improvement in hemodynamics parameters [43,44], weaning rate [43,45] and survival [45,46].

### 4.2. ECPELLA

The use of Impella in combination with VA-ECMO (also known as ECPELLA/ECMELLA) has been shown to provide improved weaning and survival rates compared to ECMO alone strategy and to established risks scores [47,48,49,50]. The addition of a continuous flow vent reduces LV volumes and pressures. The LV stroke volume progressively decreases as pump flow increases, with the raise of systemic blood pressure and reduction of LA and pulmonary capillary wedge pressures. Despite the aortic valve does not open, there is no risk of blood stasis in the LV and the aortic root. The uncoupling of LV and aortic pressure is a sign of an effective unloading of the ventricle. In this situation a flat systemic pressure line is a sign of maximal unloading. Secondary changes in myocardial contractility and peripheral resistance may further enhance the LV unloading [26,50]. The Impella can also reduce RV afterload and facilitate RV output and pulmonary blood flow with improvement in gas exchange [51,52]. Alongside these hemodynamic features, the use of an axial flow pump may provide a circulatory support while weaning from VA-ECMO. The possibility of reducing the duration of ECLS has been reported by Scharge et al. [50], however, in the experience of Pappalardo et al. [48], the association of Impella and VA-ECMO prolonged the time of support but provided a successful recovery of patients who might not have survived under VA-ECMO treatment alone. The use of Impella has been associated with a significant risk of severe bleeding, vascular complications and cerebral stroke [53,54]. In patients receiving the dual treatment with VA-ECMO, a higher occurrence of hemolysis has been reported [48], however, no difference was generally found in terms of risk of major and minor bleeding, and cerebral stroke compared to VA-ECMO alone [47,49]. These initial results seem to support an expanding use of Impella for LV unloading. Despite the evidences are still limited and coming from retrospective studies, most of the patients who underwent ECPELLA therapy were in cardiogenic shock with severely impaired LV function, were upgraded to VA-ECMO while on axial flow pump due to a progressive deterioration, or needed the implantation of Impella following significant and complicated LV distension.

### 4.3. Other Techniques

Other unloading strategies have been reported in the literature and address the endpoints of this meta-analysis (Table A5). Briefly, the left atrium can be drained surgically by a cannula in the left atrial roof or in the right superior pulmonary vein or percutaneously [32,55] by an interatrial septostomy (septostomy usually with ballooning or stent) or a cannula attached to the ECMO venous return or to device like TamdemHeart^®^). Direct left ventricle unloading can be also achieved or by a surgical cannulation of the ventricle apex [56,57] and through the mitral valve from the left atrium [56,58] or percutaneously by a catheter across the aortic valve. The surgical or percutaneous pulmonary artery cannulation [56,57], increasing the right-side blood drainage, will indirectly reduce the pulmonary venous return and left cardiac chamber loading. The experiences with these last unloading strategies include small populations, however, these studies found a positive impact of these adjuncts on patients’ survival.

Hemodynamic responses to ECMO are different among patients and are affected by clinical presentation, associated comorbidities and the cardiovascular system coupling. This high variability may explain the difficulties in driving robust conclusions in terms of efficacy and safety of LV unloading during VA-ECMO.

Up to date and to the best of our knowledge other two meta-analysis have been published on LV unloading strategy [30,37]. In 2015, Cheng et al. [30] reported the impact of IABP on survival among 1517 patients (16 studies). The cumulative survival rate for patients on ECMO was 256/683 (37.5%) compared with 294/834 (35.3%) for patients with adjunctive IABP. Concomitant IABP was not associated with improved survival (RR: 1.143; 95% CI: 0.973 to 1.343; *p* = 0.10). IABP was not associated with improved survival in AMI patients (RR, 1.120; 95% CI, 0.772–1.624; *p* = 0.55), PCS (RR, 1.121; 95% CI, 0.826–1.520; *p* = 0.46) when placed prior to ECMO initiation (RR, 0.948; 95% CI, 0.718–1.252; *p* = 0.71), or when routinely inserted (RR, 1.102; 95% CI, 0.806–1.506; *p* = 0.54). Recently, Russo et al. [37] reviewed 17 observational studies including 3997 patients. A total of 1696 (42%) patients received a concomitant left ventricular unloading strategy while on VA-ECMO (IABP 91.7%, percutaneous ventricular assist device 5.5%, pulmonary vein or transseptal left atrial cannulation 2.8%). Mortality was lower in patients with (54%) versus without (65%) left ventricular unloading while on VA-ECMO (RR: 0.79; 95% confidence interval (CI): 0.72 to 0.87; *p < 0*.00001). Bleeding, limb ischemia, renal replacement therapy, multiorgan failure and stroke or transient ischemic attack were not demonstrably different in patients treated with VA-ECMO with versus without left ventricular unloading. Hemolysis was the only secondary outcome higher in patients who underwent VA-ECMO with left ventricular unloading (RR: 2.15; 95% CI: 1.49 to 3.11; *p < 0*.0001).

### 4.4. Limitations

As analysis of only non-randomized studies, our analysis shared similar limitations with these reports which included experiences with small populations and lacked some critical information about the timing of ECMO institution, the timing of LV unloading adjunct, or the weaning protocols. Most importantly, none of the studies report exact criteria for therapy escalation e.g., addition of IABP or Impella device to ECMO. In addition, observational nature of these studies promotes selection bias. However, compared to previous meta-analyses, that present certain methodological flaws (e.g., Russo by applying the very same search strategy included 17 studies and 3997 patients), the current study, including 62 studies and more than 7500 patients, represents the first comprehensive approach addressing LV unloading strategies during ECMO support.

We found that, regardless the strategy (IABP, Impella, others) and the etiology (PCS [59,60,61], AMI, other), LV unloading has a positive impact in patients’ weaning, without adding any further risk of CVE, sepsis, acute renal injury requiring dialysis, limb complications and reoperation for bleeding. We have also provided a separate analysis of propensity-score matched and adjusted studies, trying, in the absence of prospective randomized data, to address the high heterogeneity of the included experiences due to different baseline populations’ characteristics. This further analysis confirmed these findings favoring LV unloading techniques during VA-ECMO.

Despite the expected different flow patterns and afterload increase by central and peripheral cannulation, these two strategies were not significantly associated with a higher odds ratio risk of mortality considering the adjunct or the absence of LV unloading. However, we found a tendency in the association of higher odd ratio risk and progressively higher percentage of patients receiving peripheral cannulation, this finding couples the non-significant difference in outcomes in the PCS populations that have received a central VA-ECMO in almost 30% of the cases (less than 10% in the mixed populations, 0% in AMI patients), and suggests, within the limitations of this analysis, a more pronounced positive impact of LV unloading in the peripheral VA-ECMO setting.

The analysis of weaning, additionally included as a sensitivity analysis, might give presumptive underlying evidence of true reasons for improved survival after VA-ECMO support. The possibility of providing an adequate oxygen delivery associated with the reduction of myocardial injury and the relief of pulmonary congestion, thus enhancing arterial oxygenation and reducing pulmonary complications, may explain the higher rate of survival in patients who received an adjunct treatment able to prevent or solve left ventricular distension during VA-ECMO support.

## 5. Conclusions

During veno-arterial extracorporeal membrane oxygenation, the increase of left ventricular afterload can negatively impact the recovery from cardiogenic shock. In this meta-analysis including 7581 patients on VA-ECMO support, the adjunct of left ventricular unloading was associated with 35% higher probability of weaning and 12% lower risk of mortality.

## Supplementary Materials

The supplementary materials are available online at https://www.mdpi.com/2077-0383/9/4/1039/s1.

## Appendix A

**Table A1:** PRISMA checklist.

Section/Topic	#	Checklist Item	Reported on Page #
**Title**			
Title	1	Identify the report as a systematic review, meta-analysis, or both.	1
**Abstract**			
Structured summary	2	Provide a structured summary including, as applicable: background; objectives; data sources; study eligibility criteria, participants and interventions; study appraisal and synthesis methods; results; limitations; conclusions and implications of key findings; systematic review registration number.	2
**Introduction**			
Rationale	3	Describe the rationale for the review in the context of what is already known.	3
Objectives	4	Provide an explicit statement of questions being addressed with reference to participants, interventions, comparisons, outcomes and study design (PICOS).	3
**Methods**			
Protocol and registration	5	Indicate if a review protocol exists, if and where it can be accessed (e.g., Web address), and, if available, provide registration information including registration number.	
Eligibility criteria	6	Specify study characteristics (e.g., PICOS, length of follow-up) and report characteristics (e.g., years considered, language, publication status) used as criteria for eligibility, giving rationale.	3
Information sources	7	Describe all information sources (e.g., databases with dates of coverage, contact with study authors to identify additional studies) in the search and date last searched.	3
Search	8	Present full electronic search strategy for at least one database, including any limits used, such that it could be repeated.	3
Study selection	9	State the process for selecting studies (i.e., screening, eligibility, included in systematic review, and, if applicable, included in the meta-analysis).	3
Data collection process	10	Describe method of data extraction from reports (e.g., piloted forms, independently, in duplicate) and any processes for obtaining and confirming data from investigators.	4
Data items	11	List and define all variables for which data were sought (e.g., PICOS, funding sources) and any assumptions and simplifications made.	3–4
Risk of bias in individual studies	12	Describe methods used for assessing risk of bias of individual studies (including specification of whether this was done at the study or outcome level), and how this information is to be used in any data synthesis.	4
Summary measures	13	State the principal summary measures (e.g., risk ratio, difference in means).	4
Synthesis of results	14	Describe the methods of handling data and combining results of studies, if done, including measures of consistency (e.g., I^2^) for each meta-analysis.	4
Risk of bias across studies	15	Specify any assessment of risk of bias that may affect the cumulative evidence (e.g., publication bias, selective reporting within studies).	4
Additional analyses	16	Describe methods of additional analyses (e.g., sensitivity or subgroup analyses, meta-regression), if done, indicating which were pre-specified.	4
**Result**			
Study selection	17	Give numbers of studies screened, assessed for eligibility and included in the review, with reasons for exclusions at each stage, ideally with a flow diagram.	5
Study characteristics	18	For each study, present characteristics for which data were extracted (e.g., study size, PICOS, follow-up period) and provide the citations.	22–24 + Supplementary material
Risk of bias within studies	19	Present data on risk of bias of each study and, if available, any outcome level assessment (see item 12).	Supplementary material
Results of individual studies	20	For all outcomes considered (benefits or harms), present, for each study: (a) simple summary data for each intervention group (b) effect estimates and confidence intervals, ideally with a forest plot.	Figure 2, Figure 3 and Figure 4 + Supplementary material
Synthesis of results	21	Present results of each meta-analysis done, including confidence intervals and measures of consistency.	5–8
Risk of bias across studies	22	Present results of any assessment of risk of bias across studies (see Item 15).	Supplementary material
Additional analysis	23	Give results of additional analyses, if done (e.g., sensitivity or subgroup analyses, meta-regression [see Item 16]).	5–8
**Discussion**			
Summary of evidence	24	Summarize the main findings including the strength of evidence for each main outcome; consider their relevance to key groups (e.g., healthcare providers, users and policy makers).	11–13
Limitations	25	Discuss limitations at study and outcome level (e.g., risk of bias), and at review-level (e.g., incomplete retrieval of identified research, reporting bias).	12
Conclusions	26	Provide a general interpretation of the results in the context of other evidence, and implications for future research.	11–13
**Funding**			
Funding	27	Describe sources of funding for the systematic review and other support (e.g., supply of data); role of funders for the systematic review.	13

**Table A2:** Characteristics of included studies.

Study	Setting	Unloading Strategy	Unloading Strategy Usage (%)	N. of pts	Peripheral ECMO (%)	Distal Perfusion (*n*)	ECMO Duration	Flow Rate	Total Weaning Rate (%)	Bridge to VAD (*n*)	Bridge to HTx (*n*)
Acheampong 2016	PCS	IABP	58.3	24	NR	NR	8.4 (0.8–35.4) d	NR	75	1	0
Akanni 2018	mix	Impella	12.9	225	NR	NR	3.54 (1.64–5.97) d	NR	NR	63	NR
Aoyama 2013	AMI	IABP	92.1	38	100	NR	126.5 ± 146.4 h	NR	NR	NR	NR
Asaumi 2005	Other	IABP	42.9	14	100	NR	130 (42–171) h	NR	71.4	1	0
Aso 2016	mix	IABP	36.6	1650	100	NR	2.31 d	NR	65.5	NR	NR
Aziz 2010	mix	IABP	20	10	100	10	5.8 d	3.5 to 5.0 L/min	60	1	1
Beiras-Fernandez 2011	PCS	IABP	49.3	73	NR	NR	4.4 ± 4.0 d	NR	NR	NR	NR
Beurtheret 2013	mix	IABP	31	87	100	NR	NR	NR	44.8	4	5
Biancari 2017	PCS	IABP (47); vent (5)	25.7	148	60.1	66	6.4 ± 5.6 d	NR	48.6	6	0
Brechot 2018	mix	IABP	40.2	259	100	259	2.2 ± 4.3 d	3.5 to 4.5 L/min	55.2	34	21
Carroll 2015	mix	IABP+Impella	15.4	123	75	NR	NR	NR	56.1	2	29
Chen 2005	Other	IABP	60	10	100	NR	126.2 ± 56.3 h	NR	100	1	0
Chen 2006	AMI	IABP	86.1	36	100	NR	108.5 ± 77.5 h	NR	69.4	NR	NR
Chen 2018	PCS	IABP	63.3	60	100	100	5.3 ± 2.8 d	NR	48	NR	NR
Cho 2018	AMI	IABP	4.8	42	100	NR	NR	initial of 2.2 L/min/m^2^, which was subsequently regulated to maintain a mean arterial pressure of 65 mmHg	29.3	NR	NR
Choi 2018	AMI	IABP	35.2	145	NR	21	2.0 d [IQR: 1.0–4.0]	3.3 L/min	62.8	NR	1
Chung 2011	AMI	IABP	70	20	NR	NR	3.8 ± 4.3 d	NR	70	NR	NR
Czobor 2016	mix	IABP (10); Impella (1)	44	25	100	25	NR	initiated at up to 4.5 L/min and adjusted	NR	NR	NR
Elsharkawy 2010	PCS	IABP	9.4	233	33	NR	NR	NR	NR	28	25
Formica 2010	PCS	IABP	69	42	64.3	10	7.9 ± 5.3 d	to maintain a cardiac index of 2.5 l/min/m^2^	69	NR	NR
Gass 2014	mix	IABP	41.5	135	100	NR	8.5 ± 7.1 d	2.5 to 4.0 L/min	40.7	20	0
Guihaire 2017	PCS	IABP (25); vent (13)	27.2	92	84.8	NR	6 d	NR	48	2	2
Hei 2011	PCS	IABP	16.2	68	100	68	4.75 d	40–220 mL/kg/min	76.5	8	NR
Kagawa 2012	AMI	IABP	82.6	86	100	NR	24 (8–65) h	minimum flow of 2.0 L/min	50	NR	NR
Kim 2014	AMI	IABP	75.9	58	NR	NR	68.7 ± 17.4 h	NR	41.4	NR	NR
Lee 2016	mix	IABP	8.7	23	100	NR	98 (60–192) h	3.0 to 4.0 L/min	NR	NR	NR
Lee 2017	mx	IABP	16.3	135	100	NR	99.6 ± 103.23h	adjusted to maintain a cardiac index of 2.4 L/min/m^2^	39.3	NR	NR
Li 2015	PCS	IABP	59.3	123	100	123	4.3 d	3.0 L/min	56.1	NR	NR
Lin 2016	mix	IABP	57.1	529	100	256	NR	NR	NR	2	29
Lorusso 2016	other	IABP (34); vent (13)	59.6	57	82.5	63.1	9.9 ± 19 d	NR	75.5	2	3
Luo 2009	mix	IABP	24.4	45	88.9	NR	5.48 d	Initially, 2.5 l/min/m2 with the condition improved, 40 mL/kg/min. adjusted the ECMO blood flow rate in time to maintain LVEF	60	5	NR
Mikus 2013	PCS	IABP	92.9	14	42.9	14	5 d	to maintain cardiac index of 2.6 l/min/m^2^	50	0	0
Muller 2016	AMI	IABP (96); Impella (3)	69.6	138	NR	132	7 d	NR	35.5	13	18
Nakamura 2015	other	IABP	95.5	22	100	22	179 ± 25 h	initial flow rate was 3.0–3.5 L/min; According to the indicators of peripheral circulatory failure (e.g., arterial blood gas analysis, mixed venous oxygen saturation, lactic acid and urinary output), the flow rate of ECMO was decreased	NR	1	0
Negi 2016	AMI	IABP	60	15	100	NR	1.875 d	NR	53.3	NR	1
Overtchouk 2018	AMI	IABP	59.4	106	NR	106	NR	NR	NR	10	2
Papadopoulos 2015	PCS	IABP	21.9	360	90	NR	7 ± 1 d	50-70 mL/kg/min	58.1	6	2
Pappalardo 2016	mix	Impella	21.7	157	100	39	167 (72–286) h *	Maximal speed	36.3 *	8 *	0 *
Park 2014	AMI	IABP	42.7	96	100	NR	NR	initial of 2.2 L/min/ m2 and adjusted to maintain a mean arterial pressure of 65 mm Hg	60.4	NR	NR
Patel 2018	mix	Impella	45.5	66	100	NR	NR	NR	56.1	5	NR
Pokersnik 2012	PCS	IABP	59.2	49	65.3	32	3.8 ± 3.4 d	gradually increased to 2.0 L/min/m^2^ and adjusted as necessary to maintain adequate hemodynamics and oxygen delivery.	55.1	2	0
Poptsov 2014	PCS	vent	60.9	46	100	100	NR	NR	NR	NR	NR
Raffa 2017	PCS	IABP	26.7	86	34.9	NR	5 d	NR	49	NR	NR
Rastan 2010	PCS	IABP	74.1	517	39.3	121	3.28 ± 2.85 d	NR	63.3	15	5
Ro 2013	mix	IABP	23.7	253	96.4	NR	71.0 h	NR	46.6	NR	3
Russo 2010	mix	IABP	85.7	14	57.1	253	10.2 d	NR	78.6	2	6
Sakamoto 2012	AMI	IABP	95.9	98	100	NR	68.9 ± 62.7 h	NR	55.1	0	0
Santise 2014	PCS	IABP	72.2	18	17	NR	6.7 ± 3.2 d	4164 ± 679 mL/min	72.2	NR	NR
Shinn 2009	mix	IABP	33.7	92	100	24	90.9 ± 126.0 h	NR	64.1	NR	NR
Shmack 2017	mix	vent	41.7	48	20.1	NR	6.10 ± 3.81 d	2.6 L/min/m^2^	NR	14	5
Slottosch 2012	PCS	IABP	93.5	77	100	77	79 ± 57 h	4-7 L/min	62.3	NR	NR
Slottosch 2017	mix	IABP	74.8	139	79.1	NR	4.9 d	4-7 L/min	43.2	NR	15
Smedira 2001	mix	IABP	54.5	202	75.7	NR	NR	NR	58.9	6	42
Tepper 2018	mix	IABP	50	60	0	NR	NR	5.2 L/min	60	10	NR
Unosawa 2012	PCS	IABP	83	47	68.1	NR	63.5 ± 61.5 h	2.34 L/min	61.7	0	0
van den Brink 2017	AMI	IABP	16.7	12	100	NR	5 (1–10) d	NR	66.7	1	NR
Wang 2013	PCS	IABP	47.1	87	NR	37	61 ± 37 h	calculated to supply at least adequate total systemic circulatory support (2.2 L/min) and to achieve a SvO_2_ of 70%	58.6	NR	NR
Weber 2017	mix	IABP	27.3	11	100	11	123.8 ± 120.9 h	NR	0	NR	NR
Wu 2012	mix	IABP	73.3	60	NR	NR	NR	NR	63.3	NR	NR
Xu 2016	mix	IABP	68.8	16	NR	NR	119.3 ± 114.8 h	NR	NR	NR	NR
Zhao 2015	PCS	IABP	66.7	24	95.8	NR	115.23 ± 70.17 h	49 mL/ min/kg	66.7	1*	NR
Zhong 2017	PCS	IABP (9); vent (3)	33.3	36	80.6	NR	77.5 ± 34.5 h	NR	66.7	NR	NR

* concurrent use of LVAD and ECMO.

**Table A3:** Characteristics of patients.

Study	Setting	Unloading Strategy	Unloading Strategy Usage (%)	N. of pts	Age (Years)	Male (%)	Diabetes (%)	Hypertension (%)	PCI * (%)	CABG ** (%)
Acheampong 2016	PCS	IABP	58.3	24	41 (IQR: 22–75)	58.3	NR	NR	NA	NA
Akanni 2018	mix	Impella	12.9	225	57 (46–67)	69.3	29	57	NR	NR
Aoyama 2013	AMI	IABP	92.1	38	59.9 ± 13.5	92.1	NR	NR	89	11
Asaumi 2005	Other	IABP	42.9	14	38.4 ± 15.8	50	NR	NR	NA	NA
Aso 2016	mix	IABP	36.6	1650	NR	69.4	NR	NR	NR	NR
Aziz 2010	mix	IABP	20	10	45.3 ± 18.9	50	10	40	NR	NR
Beiras-Fernandez 2011	PCS	IABP	49.3	73	49.3 ± 18.0	64.4	NR	NR	NA	NA
Beurtheret 2013	mix	IABP	31	87	46 ± 15	67.8	15	24	NR	NR
Biancari 2017	PCS	IABP (47); vent (5)	25.7	148	65.4 ± 9.4	78.4	40	NR	NA	NA
Brechot 2018	mix	IABP	40.2	259	50.2	69.9	NR	NR	NR	NR
Carroll 2015	mix	IABP+Impella	15.4	123	56 (41–65)	69	20	42	6	4
Chen 2005	Other	IABP	60	10	37.4 ± 14.7	NR	NR	NR	NA	NA
Chen 2006	AMI	IABP	86.1	36	57 ± 10	91.7	39	NR	19	78
Chen 2018	PCS	IABP	63.3	60	51.4 ± 12.7	75	17	33	NA	NA
Cho 2018	AMI	IABP	4.8	42	63.48 ± 11.46	66.7	41	48	100 [74]	0
Choi 2018	AMI	IABP	35.2	145	64.6 ± 11.7	75.9	54	53	90 [83]	NR
Chung 2011	AMI	IABP	70	20	67.7 ± 11.7	30	35	45	35	55
Czobor 2016	mix	IABP (10); Impella (1)	44	25	NR	80	44	52	NR	NR
Elsharkawy 2010	PCS	IABP	9.4	233	NR	67.4	21	NR	NA	NA
Formica 2010	PCS	IABP	69	42	64.3 ± 11.3	66.7	33	67	NA	NA
Gass 2014	mix	IABP	41.5	135	57.3 ± 15.3	64.4	31	48	NR	NR
Guihaire 2017	PCS	IABP (25); vent (13)	27.2	92	64.5 (18-83)	59	NR	NR	NA	NA
Hei 2011	PCS	IABP	16.2	68	49.2 ± 13.3	76.5	NR	NR	NA	NA
Kagawa 2012	AMI	IABP	82.6	86	63 (56–72)	81	31	63	71	0
Kim 2014	AMI	IABP	75.9	58	61.2 ± 11.3	82.8	NR	NR	NR	NR
Lee 2016	mix	IABP	8.7	23	55 (40, 68)	90	52	52	65	NR
Lee 2017	mx	IABP	16.3	135	59.44 ± 16.55	69.6	38	42	NR	NR
Li 2015	PCS	IABP	59.3	123	56.2 ± 11.8	65.9	NR	NR	NA	NA
Lin 2016	mix	IABP	57.1	529	55.1 ± 15.3	75.4	32	35	NR	NR
Lorusso 2016	other	IABP (34); vent (13)	59.6	57	37.6 ± 11.8	35.1	NR	NR	NR	NR
Luo 2009	mix	IABP	24.4	45	49.0 ± 14.1	76	NR	NR	NA	NA
Mikus 2013	PCS	IABP	92.9	14	53.1 ± 14.3	64.3	29	64	NA	NA
Muller 2016	AMI	IABP (96); Impella (3)	69.6	138	55 (46–63)	80	NR	NR	81 [72]	NR
Nakamura 2015	other	IABP	95.5	22	46.2 ± 18.7	45.5	NR	NR	NA	NA
Negi 2016	AMI	IABP	60	15	57 ± 13	60	20	87	NR	NR
Overtchouk 2018	AMI	IABP	59.4	106	52.7 ± 10.4	84	21	37	75 [72]	4
Papadopoulos 2015	PCS	IABP	21.9	360	62 ± 17	76.1	42	63	NA	NA
Pappalardo 2016	mix	Impella	21.7	157	53 (46–65)	83	NR	NR	36	NR
Park 2014	AMI	IABP	42.7	96	NR	77.1	61	48	81 [63]	10
Patel 2018	mix	Impella	45.5	66	NR	68.2	NR	NR	15	29
Pokersnik 2012	PCS	IABP	59.2	49	65 ± 13	67.3	39	90	NA	NA
Poptsov 2014	PCS	vent	60.9	46	42.1 ± 4.1	76.1	NR	NR	NA	NA
Raffa 2017	PCS	IABP	26.7	86	65 ± 11.2	65.1	17	94	NA	NA
Rastan 2010	PCS	IABP	74.1	517	63.5 ± 11.2	71.5	33	70	NA	NA
Ro 2013	mix	IABP	23.7	253	58.8 ± 15.3	60.9	23	39	NR	NR
Russo 2010	mix	IABP	85.7	14	47.8 ± 16.8	71.4	NR	NR	NR	NR
Sakamoto 2012	AMI	IABP	95.9	98	72 ± 12	66.3	35	45	94 [66]	2
Santise 2014	PCS	IABP	72.2	18	49 ± 11	77.8	17	22	NA	NA
Shinn 2009	mix	IABP	33.7	92	56 ± 18	64.1	24	29	NR	NR
Shmack 2017	mix	vent	41.7	48	49.7 ± 19.5	47.9	NR	NR	NR	NR
Slottosch 2012	PCS	IABP	93.5	77	60 ± 13	76.6	18	50	NA	NA
Slottosch 2017	mix	IABP	74.8	139	58 ± 15	76.3	27	NR	NR	NR
Smedira 2001	mix	IABP	54.5	202	55 ± 14	72	21	NR	NR	NR
Tepper 2018	mix	IABP	50	60	53.9 ± 14.9	53.3	38	53	NR	NR
Unosawa 2012	PCS	IABP	83	47	64.4 ± 12.5	74.4	38	43	NA	NA
van den Brink 2017	AMI	IABP	16.7	12	63 (47–75)	83	17	42	100	0
Wang 2013	PCS	IABP	47.1	87	65 ± 7	58.6	11	19	NA	NA
Weber 2017	mix	IABP	27.3	11	52.5 ± 16.4	81.8	NR	NR	NR	NR
Wu 2012	mix	IABP	73.3	60	49	66.7	43 ***	NR	48 ***	48 ***
Xu 2016	mix	IABP	68.8	16	62.3 ± 11.1	62.5	38	NR	NR	NR
Zhao 2015	PCS	IABP	66.7	24	59.3 ± 11.9	79.2	25	42	NA	NA
Zhong 2017	PCS	IABP (9); vent (3)	33.3	36	50.4 ± 12.2	91.7	25	81	NA	NA

* PCI as a part of managing strategy of cardiogenic shock; data presented for studies with population with acute myocardial infarction etiology; in square brackets reported is the rate of successful angioplasty. ** CABG as a part of managing strategy of cardiogenic shock; data presented for studies with population with acute myocardial infarction etiology. *** data for AMI patients only.

**Table A4:** ROBINS-I tool bias assessment.

Study	Bias Due to Confounding	Bias in Selection of Participants into the Study	Bias in Measurement of Interventions	Bias Due to Departures from Intended Interventions	Bias Due to Missing Data *	Bias in Measurement of Outcomes *	Bias in Selection of Reported Result *	Overall Bias	Cohen’s Kappa
Acheampong 2016	Serious	Critical	Serious	NA	Moderate	Moderate	Low	Serious	0.83
Akanni 2018	Moderate	Low	Low	NA	Low	Moderate	Moderate	Moderate	1
Aoyama 2013	Serious	Low	Moderate	NA	Moderate	Serious	Serious	Serious	1
Asaumi 2005	Serious	Moderate	Serious	NA	Low	Critical	Critical	Critical	0.67
Aso 2016	Moderate	Low	Critical	NA	Low	Serious	Moderate	Moderate	0.83
Aziz 2010	Serious	Low	Low	NA	Low	Moderate	Moderate	Low	0.83
Beiras-Fernandez 2011	Moderate	Low	Low	NA	Moderate	Critical	Critical	Critical	0.83
Beurtheret 2013	Serious	Moderate	Low	NA	Moderate	Low	Low	Low	1
Biancari 2017	Low	Low	Serious	NA	Moderate	Low	Low	Low	0.83
Brechot 2018	Moderate	Low	Critical	NA	Low	Critical	Critical	Critical	1
Carroll 2015	Moderate	Low	Moderate	NA	Moderate	Moderate	Moderate	Moderate	1
Chen 2005	Moderate	Moderate	Low	NA	Low	Serious	Serious	Serious	0.83
Chen 2006	Serious	Low	Low	NA	Moderate	Serious	Serious	Serious	0.83
Chen 2018	Moderate	Low	Critical	NA	Moderate	Serious	Serious	Serious	0.67
Cho 2018	Serious	Moderate	Moderate	NA	Moderate	Serious	Serious	Serious	1
Choi 2018	Serious	Low	Serious	NA	Moderate	Serious	Serious	Serious	0.83
Chung 2011	Moderate	Low	Low	NA	Moderate	Moderate	Moderate	Moderate	1
Czobor 2016	Serious	Low	Moderate	NA	Moderate	Serious	Serious	Serious	1
Elsharkawy 2010	Serious	Low	Low	NA	Moderate	Moderate	Moderate	Moderate	0.83
Formica 2010	Moderate	Moderate	Serious	NA	Moderate	Moderate	Moderate	Moderate	0.67
Gass 2014	Moderate	Low	Critical	NA	Low	Moderate	Moderate	Moderate	1
Guihaire 2017	Low	Serious	Low	NA	Moderate	Moderate	Moderate	Moderate	1
Hei 2011	Serious	Low	Low	NA	Moderate	Moderate	Low	Low	0.83
Kagawa 2012	Moderate	Serious	Serious	NA	Moderate	Serious	Serious	Serious	0.83
Kim 2014	Moderate	Moderate	Critical	NA	Moderate	Critical	Critical	Critical	0.50
Lee 2016	Serious	Moderate	Serious	NA	Moderate	Moderate	Moderate	Moderate	1
Lee 2017	Moderate	Low	Moderate	NA	Moderate	Serious	Serious	Serious	1
Li 2015	Moderate	Low	Low	NA	Moderate	Moderate	Low	Moderate	0.83
Lin 2016	Moderate	Low	Critical	NA	Low	Serious	Serious	Serious	0.831
Lorusso 2016	Low	Moderate	Critical	NA	Moderate	Moderate	Low	Moderate	0.83
Luo 2009	Moderate	Low	Low	NA	Moderate	Moderate	Low	Moderate	0.67
Mikus 2013	Serious	Low	Low	NA	Low	Moderate	Moderate	Low	0.67
Muller 2016	Serious	Low	Low	NA	Moderate	Moderate	Moderate	Moderate	0.83
Nakamura 2015	Serious	Moderate	Serious	NA	Moderate	Moderate	Moderate	Moderate	1
Negi 2016	Moderate	Low	Low	NA	Low	Moderate	Moderate	Moderate	0.83
Overtchouk 2018	Moderate	Serious	Low	NA	Moderate	Critical	Critical	Critical	1
Papadopoulos 2015	Serious	Low	Low	NA	Moderate	Moderate	Moderate	Moderate	1
Pappalardo 2016	Moderate	Low	Critical	NA	Low	Serious	Serious	Serious	0.83
Park 2014	Moderate	Moderate	Low	NA	Moderate	Moderate	Moderate	Moderate	1
Patel 2018	Serious	Low	Low	NA	Low	Moderate	Moderate	Low	0.83
Pokersnik 2012	Serious	Serious	Critical	NA	Moderate	Moderate	Moderate	Moderate	1
Poptsov 2014	Moderate	Serious	Critical	NA	Moderate	Critical	Critical	Critical	1
Raffa 2017	Moderate	Low	Serious	NA	Moderate	Moderate	Low	Moderate	0.50
Rastan 2010	Moderate	Low	Low	NA	Moderate	Moderate	Moderate	Moderate	0.83
Ro 2013	Serious	Low	Critical	NA	Low	Critical	Critical	Critical	0.83
Russo 2010	Serious	Low	Low	NA	Low	Critical	Serious	Low	0.83
Sakamoto 2012	Moderate	Low	Moderate	NA	Moderate	Serious	Serious	Moderate	0.67
Santise 2014	Moderate	Serious	Serious	NA	Moderate	Moderate	Moderate	Moderate	0.83
Shinn 2009	Moderate	Low	Critical	NA	Moderate	Serious	Serious	Serious	0.83
Shmack 2017	Serious	Low	Serious	NA	Low	Critical	Critical	Critical	1
Slottosch 2012	Low	Low	Low	NA	Moderate	Low	Low	Low	1
Slottosch 2017	Low	Low	Low	NA	Moderate	Moderate	Low	Low	0.67
Smedira 2001	Moderate	Low	Serious	NA	Moderate	Serious	Moderate	Moderate	0.83
Tepper 2018	Moderate	Low	Critical	NA	Low	Moderate	Moderate	Moderate	1
Unosawa 2012	Serious	Low	Low	NA	Moderate	Moderate	Low	Low	1
van den Brink 2017	Moderate	Low	Critical	NA	Moderate	Serious	Serious	Serious	1
Wang 2013	Moderate	Critical	Low	NA	Moderate	Low	Low	Low	0.67
Weber 2017	Low	Critical	Critical	NA	Low	Critical	Critical	Critical	0.83
Wu 2012	Serious	Low	Moderate	NA	Moderate	Moderate	Moderate	Moderate	1
Xu 2016	Moderate	Low	Critical	NA	Moderate	Serious	Serious	Serious	0.83
Zhao 2015	Serious	Critical	Critical	NA	Moderate	Moderate	Moderate	Moderate	0.83
Zhong 2017	Low	Critical	Low	NA	Moderate	Serious	Low	Low	0.50

* When multiple outcomes were reported for a study, the highest level of bias at the outcome level is reported in the table. Bias reported for comparison of peripheral vs. central extracorporeal circulation and not for a study in general.

**Table A5:** LV unloading strategy.

	LA	RSPV	Direct LV Apex	LV by RSPV	PA
Guihaire 2017	13 patients				
Biancari 2017		3 patients	1 patient		1 patient
Poptsov 2014	19 patients (percutaneous)				
Shmack 2017				29 patients	
Lorusso 2016		4 patients	4 patients		2 patients
