# Supplementary material for: Left Ventricle Unloading with Veno-Arterial Extracorporeal Membrane Oxygenation for Cardiogenic Shock. Systematic Review and Meta-Analysis
Source: J Clin Med. 2020 Apr 7;9(4):1039. doi: 10.3390/jcm9041039 (PMC7230555; doi:10.3390/jcm9041039)
Supplement: Supplementary file 1 [file jcm-09-01039-s001.pdf]

Journal of Clinical Medicine

MDPI

Supplementary Material

Part 1

1. Doll, J.A.; Sketch, M.H., Jr. ECMO and the Intraaortic Balloon Pump: In Search of the Ideal Mechanical Circulatory Support Device. The Journal of invasive cardiology 2015, 27, 459-460.
2. Dangers, L.; Brechot, N.; Schmidt, M.; Lebreton, G.; Hekimian, G.; Nieszkowska, A.; Besset, S.; Trouillet, J.L.; Chastre, J.; Leprince, P., et al. Extracorporeal Membrane Oxygenation for Acute Decompensated Heart Failure. Critical care medicine 2017, 45, 1359-1366, doi:10.1097/CCM.0000000000002485.
3. Zhao, Y.; Xing, J.; Du, Z.; Liu, F.; Jia, M.; Hou, X. Extracorporeal cardiopulmonary resuscitation for adult patients who underwent post-cardiac surgery. European journal of medical research 2015, 20, 83, doi:10.1186/s40001-015-0179-4.
4. Luo, X.J.; Wang, W.; Hu, S.S.; Sun, H.S.; Gao, H.W.; Long, C.; Song, Y.H.; Xu, J.P. Extracorporeal membrane oxygenation for treatment of cardiac failure in adult patients. Interactive cardiovascular and thoracic surgery 2009, 9, 296-300, doi:10.1510/icvts.2008.197681.
5. Park, S.J.; Kim, S.P.; Kim, J.B.; Jung, S.H.; Choo, S.J.; Chung, C.H.; Lee, J.W. Blood lactate level during extracorporeal life support as a surrogate marker for survival. The Journal of thoracic and cardiovascular surgery 2014, 148, 714-720, doi:10.1016/j.jtcvs.2014.02.078.

Part 2

1. Acheampong, B.; Johnson, J.N.; Stulak, J.M.; Dearani, J.A.; Kushwaha, S.S.; Daly, R.C.; Haile, D.T.; Schears, G.J. Postcardiotomy ECMO Support after High-risk Operations in Adult Congenital Heart Disease. Congenital heart disease 2016, 11, 751-755, doi:10.1111/chd.12396.
2. Akanni, O.J.; Takeda, K.; Truby, L.K.; Kurlansky, P.A.; Chiuzan, C.; Han, J.; Topkara, V.K.; Yuzefpolskaya, M.; Colombo, P.C.; Karmpaliotis, D., et al. EC-VAD: Combined Use of Extracorporeal Membrane Oxygenation and Percutaneous Microaxial Pump Left Ventricular Assist Device. ASAIO journal 2019, 65, 219-226, doi:10.1097/MAT.0000000000000804.
3. Aoyama, N.; Imai, H.; Kurosawa, T.; Fukuda, N.; Moriguchi, M.; Nishinari, M.; Nishii, M.; Kono, K.; Soma, K.; Izumi, T. Therapeutic strategy using extracorporeal life support, including appropriate indication, management, limitation and timing of switch to ventricular assist device in patients with acute myocardial infarction. Journal of artificial organs: the official journal of the Japanese Society for Artificial Organs 2014, 17, 33-41, doi:10.1007/s10047-013-0735-z.
4. Asaumi, Y.; Yasuda, S.; Morii, I.; Kakuchi, H.; Otsuka, Y.; Kawamura, A.; Sasako, Y.; Nakatani, T.; Nonogi, H.; Miyazaki, S. Favourable clinical outcome in patients with cardiogenic shock due to fulminant myocarditis supported by percutaneous extracorporeal membrane oxygenation. European heart journal 2005, 26, 2185-2192, doi:10.1093/eurheartj/ehi411.
5. Aso, S.; Matsui, H.; Fushimi, K.; Yasunaga, H. The Effect of Intraaortic Balloon Pumping Under Venoarterial Extracorporeal Membrane Oxygenation on Mortality of Cardiogenic Patients: An Analysis Using a Nationwide Inpatient Database. Critical care medicine 2016, 44, 1974-1979, doi:10.1097/CCM.0000000000001828.
6. Aziz, T.A.; Singh, G.; Popjes, E.; Stephenson, E.; Mulvey, S.; Pae, W.; El-Banayosy, A. Initial experience with CentriMag extracorporal membrane oxygenation for support of critically ill patients with refractory cardiogenic shock. The Journal of heart and lung transplantation: the official publication of the International Society for Heart Transplantation 2010, 29, 66-71, doi:10.1016/j.healun.2009.08.025.
7. Beiras-Fernandez, A.; Deutsch, M.A.; Kainzinger, S.; Kaczmarek, I.; Sodian, R.; Ueberfuhr, P.; Meiser, B.; Schmoeckel, M.; Reichart, B.; Brenner, P. Extracorporeal membrane oxygenation in 108 patients with low cardiac output - a single-center experience. The International journal of artificial organs 2011, 34, 365-373, doi:10.5301/IJAO.2011.7727.

8. Beurtheret, S.; Mordant, P.; Paoletti, X.; Marijon, E.; Celermajer, D.S.; Leger, P.; Pavie, A.; Combes, A.; Leprince, P. Emergency circulatory support in refractory cardiogenic shock patients in remote institutions: a pilot study (the cardiac-RESCUE program). European heart journal 2013, 34, 112-120, doi:10.1093/eurheartj/ehs081.
9. Biancari, F.; Dalen, M.; Perrotti, A.; Fiore, A.; Reichart, D.; Khodabandeh, S.; Gulbins, H.; Zipfel, S.; Al Shakaki, M.; Welp, H., et al. Venoarterial extracorporeal membrane oxygenation after coronary artery bypass grafting: Results of a multicenter study. International journal of cardiology 2017, 241, 109-114, doi:10.1016/j.ijcard.2017.03.120.
10. Brechot, N.; Mastroianni, C.; Schmidt, M.; Santi, F.; Lebreton, G.; Hoareau, A.M.; Luyt, C.E.; Chommeloux, J.; Rigolet, M.; Lebbah, S., et al. Retrieval of severe acute respiratory failure patients on extracorporeal membrane oxygenation: Any impact on their outcomes? The Journal of thoracic and cardiovascular surgery 2018, 155, 1621-1629 e1622, doi:10.1016/j.jtcvs.2017.10.084.
11. Carroll, B.J.; Shah, R.V.; Murthy, V.; McCullough, S.A.; Reza, N.; Thomas, S.S.; Song, T.H.; Newton-Cheh, C.H.; Camuso, J.M.; MacGillivray, T., et al. Clinical Features and outcomes in adults with cardiogenic shock supported by extracorporeal membrane oxygenation. The American journal of cardiology 2015, 116, 1624-1630, doi:10.1016/j.amjcard.2015.08.030.
12. Chen, Y.S.; Yu, H.Y.; Huang, S.C.; Chiu, K.M.; Lin, T.Y.; Lai, L.P.; Lin, F.Y.; Wang, S.S.; Chu, S.H.; Ko, W.J. Experience and result of extracorporeal membrane oxygenation in treating fulminant myocarditis with shock: what mechanical support should be considered first? The Journal of heart and lung transplantation: the official publication of the International Society for Heart Transplantation 2005, 24, 81-87, doi:10.1016/j.healun.2003.09.038.
13. Chen, J.S.; Ko, W.J.; Yu, H.Y.; Lai, L.P.; Huang, S.C.; Chi, N.H.; Tsai, C.H.; Wang, S.S.; Lin, F.Y.; Chen, Y.S. Analysis of the outcome for patients experiencing myocardial infarction and cardiopulmonary resuscitation refractory to conventional therapies necessitating extracorporeal life support rescue. Critical care medicine 2006, 34, 950-957, doi:10.1097/01.CCM.0000206103.35460.1F.
14. Chen, K.; Hou, J.; Tang, H.; Hu, S. Concurrent Implantation of Intra-Aortic Balloon Pump and Extracorporeal Membrane Oxygenation Improved Survival of Patients With Postcardiotomy Cardiogenic Shock. Artificial organs 2019, 43, 142-149, doi:10.1111/aor.13317.
15. Cho, S.; Lee, W.; Lim, S.H.; Kang, T.S. Relationship between Clinical Outcomes and Cardiopulmonary Resuscitation Time in Patients with Acute Myocardial Infarction Treated by Extracorporeal Membrane Oxygenation-Assisted Primary Percutaneous Coronary Intervention. Korean circulation journal 2018, 48, 705-715, doi:10.4070/kcj.2018.0121.
16. Choi, K.H.; Yang, J.H.; Park, T.K.; Lee, J.M.; Song, Y.B.; Hahn, J.Y.; Choi, S.H.; Choi, J.H.; Cho, Y.H.; Sung, K., et al. Risk Prediction Model of In-hospital Mortality in Patients With Myocardial Infarction Treated With Venoarterial Extracorporeal Membrane Oxygenation. Revista espanola de cardiologia 2019, 72, 724-731, doi:10.1016/j.rec.2018.06.010.
17. Chung, E.S.; Lim, C.; Lee, H.Y.; Choi, J.H.; Lee, J.S.; Park, K.H. Results of Extracorporeal Membrane Oxygenation (ECMO) Support before Coronary Reperfusion in Cardiogenic Shock with Acute Myocardial Infarction. The Korean journal of thoracic and cardiovascular surgery 2011, 44, 273-278, doi:10.5090/kjtcs.2011.44.4.273.
18. Czobor, P.; Venturini, J.M.; Parikh, K.S.; Retzer, E.M.; Friant, J.; Jeevanandam, V.; Russo, M.J.; Uriel, N.; Paul, J.D.; Blair, J.E., et al. Sequential Organ Failure Assessment Score at Presentation Predicts Survival in Patients Treated With Percutaneous Veno-Arterial Extracorporeal Membrane Oxygenation. The Journal of invasive cardiology 2016, 28, 133-138.
19. Elsharkawy, H.A.; Li, L.; Esa, W.A.; Sessler, D.I.; Bashour, C.A. Outcome in patients who require venoarterial extracorporeal membrane oxygenation support after cardiac surgery. Journal of cardiothoracic and vascular anesthesia 2010, 24, 946-951, doi:10.1053/j.jvca.2010.03.020.

20. Formica, F.; Avalli, L.; Colagrande, L.; Ferro, O.; Greco, G.; Maggioni, E.; Paolini, G. Extracorporeal membrane oxygenation to support adult patients with cardiac failure: predictive factors of 30-day mortality. Interactive cardiovascular and thoracic surgery 2010, 10, 721-726, doi:10.1510/icvts.2009.220335.
21. Gass, A.; Palaniswamy, C.; Aronow, W.S.; Kolte, D.; Khera, S.; Ahmad, H.; Cuomo, L.J.; Timmermans, R.; Cohen, M.; Tang, G.H., et al. Peripheral venoarterial extracorporeal membrane oxygenation in combination with intra-aortic balloon counterpulsation in patients with cardiovascular compromise. Cardiology 2014, 129, 137-143, doi:10.1159/000365138.
22. Guihaire, J.; Dang Van, S.; Rouze, S.; Rosier, S.; Roisne, A.; Langanay, T.; Corbineau, H.; Verhoye, J.P.; Flecher, E. Clinical outcomes in patients after extracorporeal membrane oxygenation support for post-cardiotomy cardiogenic shock: a single-centre experience of 92 cases. Interactive cardiovascular and thoracic surgery 2017, 25, 363-369, doi:10.1093/icvts/ivx155.
23. Hei, F.; Lou, S.; Li, J.; Yu, K.; Liu, J.; Feng, Z.; Zhao, J.; Hu, S.; Xu, J.; Chang, Q., et al. Five-year results of 121 consecutive patients treated with extracorporeal membrane oxygenation at Fu Wai Hospital. Artificial organs 2011, 35, 572-578, doi:10.1111/j.1525-1594.2010.01151.x.
24. Kagawa, E.; Dote, K.; Kato, M.; Sasaki, S.; Nakano, Y.; Kajikawa, M.; Higashi, A.; Itakura, K.; Sera, A.; Inoue, I., et al. Should we emergently revascularize occluded coronaries for cardiac arrest?: rapid-response extracorporeal membrane oxygenation and intra-arrest percutaneous coronary intervention. Circulation 2012, 126, 1605-1613, doi:10.1161/CIRCULATIONAHA.111.067538.
25. Kim, D.K.; Seo, G.W.; Song, P.S.; Kim, K.H.; Kim, D.I.; Jin, H.Y.; Jang, J.S.; Yoon, H.J.; Nam, C.W. Impact of concomitant use of intra-aortic balloon pump during percutaneous cardiopulmonary support in patients with cardiogenic shock complicating acute myocardial infarction. Eurointervention (EuroPCR Abstracts and Poster 2014), Poster text.
26. Lee, J.J.; Han, S.J.; Kim, H.S.; Hong, K.S.; Choi, H.H.; Park, K.T.; Seo, J.Y.; Lee, T.H.; Kim, H.C.; Kim, S., et al. Out-of-hospital cardiac arrest patients treated with cardiopulmonary resuscitation using extracorporeal membrane oxygenation: focus on survival rate and neurologic outcome. Scandinavian journal of trauma, resuscitation and emergency medicine 2016, 24, 74, doi:10.1186/s13049-016-0266-8.
27. Lee, W.; Kim, Y.; Choi, H.; Kim, H.; Lee, S.; Lee, H.; Chee, H.; Kim, J.; Hwang, J.; Lee, S., et al. Advanced Age as a Predictor of Survival and Weaning in Venoarterial Extracorporeal Oxygenation: A Retrospective Observational Study. BioMed research international 2017, 2017, 3505784, doi:10.1155/2017/3505784.
28. Li, C.L.; Wang, H.; Jia, M.; Ma, N.; Meng, X.; Hou, X.T. The early dynamic behavior of lactate is linked to mortality in postcardiotomy patients with extracorporeal membrane oxygenation support: A retrospective observational study. The Journal of thoracic and cardiovascular surgery 2015, 149, 1445-1450, doi:10.1016/j.jtcvs.2014.11.052.
29. Lin, L.Y.; Liao, C.W.; Wang, C.H.; Chi, N.H.; Yu, H.Y.; Chou, N.K.; Hwang, J.J.; Lin, J.L.; Chiang, F.T.; Chen, Y.S. Effects of Additional Intra-aortic Balloon Counter-Pulsation Therapy to Cardiogenic Shock Patients Supported by Extra-corporeal Membranous Oxygenation. Scientific reports 2016, 6, 23838, doi:10.1038/srep23838.
30. Lorusso, R.; Centofanti, P.; Gelsomino, S.; Barili, F.; Di Mauro, M.; Orlando, P.; Botta, L.; Milazzo, F.; Actis Dato, G.; Casabona, R., et al. Venoarterial Extracorporeal Membrane Oxygenation for Acute Fulminant Myocarditis in Adult Patients: A 5-Year Multi-Institutional Experience. The Annals of thoracic surgery 2016, 101, 919-926, doi:10.1016/j.athoracsur.2015.08.014.
31. Luo, X.J.; Wang, W.; Hu, S.S.; Sun, H.S.; Gao, H.W.; Long, C.; Song, Y.H.; Xu, J.P. Extracorporeal membrane oxygenation for treatment of cardiac failure in adult patients. Interactive cardiovascular and thoracic surgery 2009, 9, 296-300, doi:10.1510/icvts.2008.197681.
32. Mikus, E.; Tripodi, A.; Calvi, S.; Giglio, M.D.; Cavallucci, A.; Lamarra, M. CentriMag venoarterial extracorporeal membrane oxygenation support as treatment for patients with refractory postcardiotomy cardiogenic shock. ASAIO journal 2013, 59, 18-23, doi:10.1097/MAT.0b013e3182768b68.

33. Muller, G.; Flecher, E.; Lebreton, G.; Luyt, C.E.; Trouillet, J.L.; Brechot, N.; Schmidt, M.; Mastroianni, C.; Chastre, J.; Leprince, P., et al. The ENCOURAGE mortality risk score and analysis of long-term outcomes after VA-ECMO for acute myocardial infarction with cardiogenic shock. Intensive care medicine 2016, 42, 370-378, doi:10.1007/s00134-016-4223-9.
34. Nakamura, T.; Ishida, K.; Taniguchi, Y.; Nakagawa, T.; Seguchi, M.; Wada, H.; Sugawara, Y.; Funayama, H.; Mitsuhashi, T.; Momomura, S. Prognosis of patients with fulminant myocarditis managed by peripheral venoarterial extracorporeal membranous oxygenation support: a retrospective single-center study. Journal of intensive care 2015, 3, 5, doi:10.1186/s40560-014-0069-9.
35. Negi, S.I.; Sokolovic, M.; Koifman, E.; Kiramijyan, S.; Torguson, R.; Lindsay, J.; Ben-Dor, I.; Suddath, W.; Pichard, A.; Satler, L., et al. Contemporary Use of Veno-Arterial Extracorporeal Membrane Oxygenation for Refractory Cardiogenic Shock in Acute Coronary Syndrome. The Journal of invasive cardiology 2016, 28, 52-57.
36. Overtchouk, P.; Pascal, J.; Lebreton, G.; Hulot, J.S.; Luyt, C.E.; Combes, A.; Kerneis, M.; Silvain, J.; Barthelemy, O.; Leprince, P., et al. Outcome after revascularisation of acute myocardial infarction with cardiogenic shock on extracorporeal life support. EuroIntervention: journal of EuroPCR in collaboration with the Working Group on Interventional Cardiology of the European Society of Cardiology 2018, 13, e2160-e2168, doi:10.4244/EIJ-D-17-01014.
37. Papadopoulos, N.; Marinos, S.; El-Sayed Ahmad, A.; Keller, H.; Meybohm, P.; Zacharowski, K.; Moritz, A.; Zierer, A. Risk factors associated with adverse outcome following extracorporeal life support: analysis from 360 consecutive patients. Perfusion 2015, 30, 284-290, doi:10.1177/0267659114542458.
38. Pappalardo, F.; Schulte, C.; Pieri, M.; Schrage, B.; Contri, R.; Soeffker, G.; Greco, T.; Lembo, R.; Mullerleile, K.; Colombo, A., et al. Concomitant implantation of Impella((R)) on top of veno-arterial extracorporeal membrane oxygenation may improve survival of patients with cardiogenic shock. European journal of heart failure 2017, 19, 404-412, doi:10.1002/ejhf.668.
39. Park, T.K.; Yang, J.H.; Choi, S.H.; Song, Y.B.; Hahn, J.Y.; Choi, J.H.; Sung, K.; Lee, Y.T.; Gwon, H.C.; Lee, S.H. Clinical outcomes of patients with acute myocardial infarction complicated by severe refractory cardiogenic shock assisted with percutaneous cardiopulmonary support. Yonsei medical journal 2014, 55, 920-927, doi:10.3349/ymj.2014.55.4.920.
40. Patel, S.M.; Lipinski, J.; Al-Kindi, S.G.; Patel, T.; Saric, P.; Li, J.; Nadeem, F.; Ladas, T.; Alaiti, A.; Phillips, A., et al. Simultaneous Venoarterial Extracorporeal Membrane Oxygenation and Percutaneous Left Ventricular Decompression Therapy with Impella Is Associated with Improved Outcomes in Refractory Cardiogenic Shock. ASAIO journal 2019, 65, 21-28, doi:10.1097/MAT.0000000000000767.
41. Pokersnik, J.A.; Buda, T.; Bashour, C.A.; Gonzalez-Stawinski, G.V. Have changes in ECMO technology impacted outcomes in adult patients developing postcardiotomy cardiogenic shock? Journal of cardiac surgery 2012, 27, 246-252, doi:10.1111/j.1540-8191.2011.01409.x.
42. Poptsov, V.; Spirina, E.; Dogonasheva, A.; Zolotova, E. Five years' experience with a peripheral veno-arterial ECMO for mechanical bridge to heart transplantation. Journal of thoracic disease 2019, 11, S889-S901, doi:10.21037/jtd.2019.02.55.???
43. Raffa, G.M.; Gelsomino, S.; Sluijpers, N.; Meani, P.; Alenizy, K.; Natour, E.; Bidar, E.; Johnson, D.M.; Makhoul, M.; Heuts, S., et al. In-hospital outcome of post-cardiotomy extracorporeal life support in adult patients: the 2007-2017 Maastricht experience. Critical care and resuscitation: journal of the Australasian Academy of Critical Care Medicine 2017, 19, 53-61.
44. Rastan, A.J.; Dege, A.; Mohr, M.; Doll, N.; Falk, V.; Walther, T.; Mohr, F.W. Early and late outcomes of 517 consecutive adult patients treated with extracorporeal membrane oxygenation for refractory postcardiotomy cardiogenic shock. The Journal of thoracic and cardiovascular surgery 2010, 139, 302-311, 311 e301, doi:10.1016/j.jtcvs.2009.10.043.

45. Ro, S.K.; Kim, J.B.; Jung, S.H.; Choo, S.J.; Chung, C.H.; Lee, J.W. Extracorporeal life support for cardiogenic shock: influence of concomitant intra-aortic balloon counterpulsation. European journal of cardio-thoracic surgery: official journal of the European Association for Cardio-thoracic Surgery 2014, 46, 186-192; discussion 192, doi:10.1093/ejcts/ezu005.
46. Russo, C.F.; Cannata, A.; Lanfranconi, M.; Bruschi, G.; Milazzo, F.; Paino, R.; Martinelli, L. Veno-arterial extracorporeal membrane oxygenation using Levitronix centrifugal pump as bridge to decision for refractory cardiogenic shock. The Journal of thoracic and cardiovascular surgery 2010, 140, 1416-1421, doi:10.1016/j.jtcvs.2010.07.083.
47. Sakamoto, S.; Taniguchi, N.; Nakajima, S.; Takahashi, A. Extracorporeal life support for cardiogenic shock or cardiac arrest due to acute coronary syndrome. The Annals of thoracic surgery 2012, 94, 1-7, doi:10.1016/j.athoracsur.2012.01.032.
48. Santise, G.; Panarello, G.; Ruperto, C.; Turrisi, M.; Pilato, G.; Giunta, A.; Sciacca, S.; Pilato, M. Extracorporeal membrane oxygenation for graft failure after heart transplantation: a multidisciplinary approach to maximize weaning rate. The International journal of artificial organs 2014, 37, 706-714, doi:10.5301/ijao.5000353.
49. Shinn, S.H.; Lee, Y.T.; Sung, K.; Min, S.; Kim, W.S.; Park, P.W.; Ha, Y.K. Efficacy of emergent percutaneous cardiopulmonary support in cardiac or respiratory failure: fight or flight? Interactive cardiovascular and thoracic surgery 2009, 9, 269-273, doi:10.1510/icvts.2008.194860.
50. Schmack, B.; Seppelt, P.; Weymann, A.; Alt, C.; Farag, M.; Arif, R.; Doesch, A.O.; Raake, P.W.; Kallenbach, K.; Mansur, A., et al. Extracorporeal life support with left ventricular decompression-improved survival in severe cardiogenic shock: results from a retrospective study. PeerJ 2017, 5, e3813, doi:10.7717/peerj.3813.
51. Slottosch, I.; Liakopoulos, O.; Kuhn, E.; Deppe, A.C.; Scherner, M.; Madershahian, N.; Choi, Y.H.; Wahlers, T. Outcomes after peripheral extracorporeal membrane oxygenation therapy for postcardiotomy cardiogenic shock: a single-center experience. The Journal of surgical research 2013, 181, e47-55, doi:10.1016/j.jss.2012.07.030.
52. Slottosch, I.; Liakopoulos, O.; Kuhn, E.; Scherner, M.; Deppe, A.C.; Sabashnikov, A.; Mader, N.; Choi, Y.H.; Wippermann, J.; Wahlers, T. Lactate and lactate clearance as valuable tool to evaluate ECMO therapy in cardiogenic shock. Journal of critical care 2017, 42, 35-41, doi:10.1016/j.jcrc.2017.06.022.
53. Smedira, N.G.; Moazami, N.; Golding, C.M.; McCarthy, P.M.; Apperson-Hansen, C.; Blackstone, E.H.; Cosgrove, D.M., 3rd. Clinical experience with 202 adults receiving extracorporeal membrane oxygenation for cardiac failure: survival at five years. The Journal of thoracic and cardiovascular surgery 2001, 122, 92-102, doi:10.1067/mtc.2001.114351.
54. Tepper, S.; Garcia, M.B.; Fischer, I.; Ahmed, A.; Khan, A.; Balsara, K.R.; Masood, M.F.; Itoh, A. Clinical Outcomes and Reduced Pulmonary Artery Pressure With Intra-Aortic Balloon Pump During Central Extracorporeal Life Support. ASAIO journal 2019, 65, 173-179, doi:10.1097/MAT.0000000000000788.
55. Unosawa, S.; Sezai, A.; Hata, M.; Nakata, K.; Yoshitake, I.; Wakui, S.; Kimura, H.; Takahashi, K.; Hata, H.; Shiono, M. Long-term outcomes of patients undergoing extracorporeal membrane oxygenation for refractory postcardiotomy cardiogenic shock. Surgery today 2013, 43, 264-270, doi:10.1007/s00595-012-0322-6.
56. van den Brink, F.S.; Magan, A.D.; Noordzij, P.G.; Zivelonghi, C.; Agostoni, P.; Eefting, F.D.; Ten Berg, J.M.; Suttorp, M.J.; Rensing, B.R.; van Kuijk, J.P., et al. Veno-arterial extracorporeal membrane oxygenation in addition to primary PCI in patients presenting with ST-elevation myocardial infarction. Netherlands heart journal: monthly journal of the Netherlands Society of Cardiology and the Netherlands Heart Foundation 2018, 26, 76-84, doi:10.1007/s12471-017-1068-y.
57. Wang, J.G.; Han, J.; Jia, Y.X.; Zeng, W.; Hou, X.T.; Meng, X. Outcome of veno-arterial extracorporeal membrane oxygenation for patients undergoing valvular surgery. PloS one 2013, 8, e63924, doi:10.1371/journal.pone.0063924.

58. Weber, C.; Deppe, A.C.; Sabashnikov, A.; Slottosch, I.; Kuhn, E.; Eghbalzadeh, K.; Scherner, M.; Choi, Y.H.; Madershahian, N.; Wahlers, T. Left ventricular thrombus formation in patients undergoing femoral veno-arterial extracorporeal membrane oxygenation. Perfusion 2018, 33, 283-288, doi:10.1177/0267659117745369.
59. Wu, M.Y.; Lee, M.Y.; Lin, C.C.; Chang, Y.S.; Tsai, F.C.; Lin, P.J. Resuscitation of non-postcardiotomy cardiogenic shock or cardiac arrest with extracorporeal life support: the role of bridging to intervention. Resuscitation 2012, 83, 976-981, doi:10.1016/j.resuscitation.2012.01.010.
60. Xu, J.; Leung, D.; Rajaratnam, R.; Mussap, C.; French, J.; Juergens, C.; Parr, M.; Lo, S.; Contemporary Practice of Veno-Arterial Extracorporeal Membrane Oxygenation (VA-ECMO) at a Single Non-Transplant Centre: Survival Prediction Scores, Indications and Outcomes. 2016; 25(Supplement 2): S171, doi:10.1016/j.hlc.2016.06.402
61. Zhao, Y.; Xing, J.; Du, Z.; Liu, F.; Jia, M.; Hou, X. Extracorporeal cardiopulmonary resuscitation for adult patients who underwent post-cardiac surgery. European journal of medical research 2015, 20, 83, doi:10.1186/s40001-015-0179-4.
62. Zhong, Z.; Jiang, C.; Yang, F.; Hao, X.; Xing, J.; Wang, H.; Hou, X. Veno-Arterial Extracorporeal Membrane Oxygenation Support in Patients Undergoing Aortic Surgery. Artificial organs 2017, 41, 1113-1120, doi:10.1111/aor.12951

Part 3

1. Aso, S.; Matsui, H.; Fushimi, K.; Yasunaga, H. The Effect of Intraaortic Balloon Pumping Under Venoarterial Extracorporeal Membrane Oxygenation on Mortality of Cardiogenic Patients: An Analysis Using a Nationwide Inpatient Database. Critical care medicine 2016, 44, 1974-1979, doi:10.1097/CCM.0000000000001828.
2. Brechot, N.; Mastroianni, C.; Schmidt, M.; Santi, F.; Lebreton, G.; Hoareau, A.M.; Luyt, C.E.; Chommeloux, J.; Rigolet, M.; Lebbah, S., et al. Retrieval of severe acute respiratory failure patients on extracorporeal membrane oxygenation: Any impact on their outcomes? The Journal of thoracic and cardiovascular surgery 2018, 155, 1621-1629 e1622, doi:10.1016/j.jtcvs.2017.10.084.
3. Lin, L.Y.; Liao, C.W.; Wang, C.H.; Chi, N.H.; Yu, H.Y.; Chou, N.K.; Hwang, J.J.; Lin, J.L.; Chiang, F.T.; Chen, Y.S. Effects of Additional Intra-aortic Balloon Counter-Pulsation Therapy to Cardiogenic Shock Patients Supported by Extra-corporeal Membranous Oxygenation. Scientific reports 2016, 6, 23838, doi:10.1038/srep23838.
4. Pappalardo, F.; Schulte, C.; Pieri, M.; Schrage, B.; Contri, R.; Soeffker, G.; Greco, T.; Lembo, R.; Mullerleile, K.; Colombo, A., et al. Concomitant implantation of Impella((R)) on top of veno-arterial extracorporeal membrane oxygenation may improve survival of patients with cardiogenic shock. European journal of heart failure 2017, 19, 404-412, doi:10.1002/ejhf.668.
5. Ro, S.K.; Kim, J.B.; Jung, S.H.; Choo, S.J.; Chung, C.H.; Lee, J.W. Extracorporeal life support for cardiogenic shock: influence of concomitant intra-aortic balloon counterpulsation. European journal of cardio-thoracic surgery: official journal of the European Association for Cardio-thoracic Surgery 2014, 46, 186-192; discussion 192, doi:10.1093/ejcts/ezu005.

Part 4

1. Aso, S.; Matsui, H.; Fushimi, K.; Yasunaga, H. The Effect of Intraaortic Balloon Pumping Under Venoarterial Extracorporeal Membrane Oxygenation on Mortality of Cardiogenic Patients: An Analysis Using a Nationwide Inpatient Database. Critical care medicine 2016, 44, 1974-1979, doi:10.1097/CCM.0000000000001828.
2. Brechot, N.; Mastroianni, C.; Schmidt, M.; Santi, F.; Lebreton, G.; Hoareau, A.M.; Luyt, C.E.; Chommeloux, J.; Rigolet, M.; Lebbah, S., et al. Retrieval of severe acute respiratory failure patients on extracorporeal membrane oxygenation: Any impact on their outcomes? The Journal of thoracic and cardiovascular surgery 2018, 155, 1621-1629 e1622, doi:10.1016/j.jtcvs.2017.10.084.
3. Pappalardo, F.; Schulte, C.; Pieri, M.; Schrage, B.; Contri, R.; Soeffker, G.; Greco, T.; Lembo, R.; Mullerleile, K.; Colombo, A., et al. Concomitant implantation of Impella((R)) on top of veno-arterial extracorporeal membrane oxygenation may improve survival of patients with cardiogenic shock. European journal of heart failure 2017, 19, 404-412, doi:10.1002/ejhf.668.
4. Ro, S.K.; Kim, J.B.; Jung, S.H.; Choo, S.J.; Chung, C.H.; Lee, J.W. Extracorporeal life support for cardiogenic shock: influence of concomitant intra-aortic balloon counterpulsation. European journal of cardio-thoracic surgery: official journal of the European Association for Cardio-thoracic Surgery 2014, 46, 186-192; discussion 192, doi:10.1093/ejcts/ezu005.

© 2020 by the authors. Licensee MDPI, Basel, Switzerland. This article is an open access article distributed under the terms and conditions of the Creative Commons Attribution (CC BY) license (http://creativecommons.org/licenses/by/4.0/).
